# Systematic pan-cancer analysis of the nicotinamide n-methyltransferase in human cancer

**DOI:** 10.3389/fgene.2022.1000515

**Published:** 2022-10-26

**Authors:** Li Cao, Wei Wu, Xiangyu Deng, Yizhong Peng, Yangyang Chen, Haoyu Guo, Lutong Wang, Xingyin Li, Zhicai Zhang, Zengwu Shao

**Affiliations:** Department of Orthopaedic, Union Hospital, Tongji Medical College, Huazhong University of Science and Technology, Wuhan, China

**Keywords:** NNMT, pan-cancer analysis, tumor, prognosis, immune infiltration, biomarker

## Abstract

In several tumors, Nicotinamide N-Methyltransferase (NNMT) was identified as a bridge between methylation metabolism and tumorigenesis and was associated with a poor prognosis. This research aims is to study the prognostic value of NNMT in cancer, its relationship with DNA methylation, and the immune microenvironment. On the basis of the Cancer Genome Atlas and the Cancer Cell Line Encyclopedia, Genotype Tissue-Expression, cBioPortal, Cellminer, Gene Expression Profiling Interactive Analysis, Human Protein Atlas and Clinical Proteomic Tumor Analysis Consortium, we used a series of bioinformatics strategies to investigate the potential carcinogenicity of NNMT, including the relationship between NNMT expression and prognosis, tumor mutational burden, microsatellite instability, and sensitivity analysis of anticancer drugs. The GeneMANIA, STRING, and BioGRID databases were examined for protein-protein interactions, and Gene Ontology and the Kyoto Encyclopedia of Genes were used to infer the signal pathway. The results indicated that NNMT was significantly expressed in several tumor tissues compared to the matching non-tumor tissues. Increased NNMT expression was linked to reduced OS, DSS, and DFI. In addition, there was a link between NNMT expression and TMB and MSI in 18 cancer types, and between NNMT expression and DNA methylation in 23 cancer types. Further study of NNMT gene alteration data revealed that deletion was the most prevalent form of NNMT mutation, and that there was a significant negative association between NNMT expression and mismatch repair genes. In addition, there was a strong positive connection between NNMT and immune infiltration in 28 types of tumors, and the immune cells that infiltrated the tumors displayed a characteristic NNMT pattern. According to the enrichment study, cell migration, cell motility, and cell adhesion were highly enriched in biological processes, and NNMT may be associated with the PI3K-Akt signaling pathway. By downregulating gene methylation or impacting the immunological microenvironment widely, NNMT may drive carcinogenesis and cause a poor prognosis. Our research showed that NNMT could be used as a biomarker of tumor immune infiltration and poor prognosis, thus providing a unique strategy for cancer therapy.

## Introduction

Nicotinamide-N-methyltransferase (NNMT), also known as EC 2.1.1.1, is a catalytic enzyme that transfers methyl groups from S-adenosylmethionine (SAM) to other substrates, producing S-adenosyl-homocysteine and methylated substrates. It contributes to cancer and development by modulating metabolic reprogramming and epigenetic changes (Lu and Long, 2018; Parsons and Facey, 2021). Mammal NNMT proteins are highly conserved and catalyze the N-methylation of NAM by depleting SAM, thereby regulating metabolic processes utilizing NAM and SAM. NNMT is implicated in NAD + -related signaling pathways, the cycling of folate and methionine, and chromatin remodeling (Schmeisser et al., 2013; Cantó et al., 2015). In the past, it was believed that NNMT only had a function in facilitating the excretion of substrates *via* methylation. However, new investigations have revealed that the expression of NNMT is markedly elevated in a variety of cancers. High NNMT expression was shown to be an unfavorable postoperative prognosis in breast cancer (Wang et al., 2019), head and neck squamous cell carcinoma (Togni et al., 2021), renal cancer (Qu et al., 2022; Reustle et al., 2022), non-small cell lung cancer (Wang et al., 2022), colorectal cancer (Song et al., 2020), pancreatic cancer (Xu et al., 2016), liver cancer (Kim et al., 2009), gastric cancer (Chen et al., 2016), ovarian cancer (Eckert et al., 2019) and endometrial cancer (Akar et al., 2019). NNMT is currently a possible biomarker and a crucial mediator in a number of human malignancies, although the relationship between NNMT activity and tumorigenesis is still unknown. In short, mounting data reveals that NNMT is an emerging regulatory component in malignancies, and that its expression can influence the prognosis of the patient.

It is now widely known that tumor cells can reshape their surrounding niche into a favorable environment to better meet their growth and dissemination needs as well as their response to hostile conditions such as oxygen deficiency, nutrient deficiency, waste accumulation, acidity, chemotherapy, etc. (Hui and Chen, 2015; Jahanban-Esfahlan et al., 2017). Notwithstanding the identification of oncogenes, tumor suppressor genes, and immune checkpoint genes, which are linked to signaling pathways that regulate cell survival or metabolic reprogramming in order to improve drug molecules and therapy techniques, there have been obstacles in the treatment of cancer (Pitt et al., 2016). Tumor Microenvironment (TME) is the cellular environment supporting tumorigenesis, composed of surrounding blood vessels, the extracellular matrix (ECM), signaling molecules, and non-malignant cells like stromal cells, fibroblasts, immune cells (such as T lymphocytes, B lymphocytes, natural killer cells and natural killer T cells, Tumor endothelial cells, Tumor-associated macrophages, etc.), and pericytes (Baghban et al., 2020). TME promotes angiogenesis, reprograms tumor stroma, therapeutic resistance, migration, invasion, and epithelial-mesenchymal transition (EMT) *via* genetic, metabolic, and epigenetic processes during tumor development (Hui and Chen, 2015; Indini et al., 2022). A thorough evaluation of the epigenetic or metabolic reprogramming genes in tumors may help the development of immunotherapies that eradicate TME barry, as immunotherapy is still one of the most essential and effective approaches to treating cancer (Pitt et al., 2016). As a result, it is vital to assess the function of the interaction between the central regulator and the immune system in order to verify potential immune-related therapeutic biomarkers in malignancies.

To the best of our knowledge, the majority of research on the role of NNMT in tumors has been limited to a single type of cancer. No pan-cancer research investigating the connection between NNMT and various cancers has been conducted. Therefore, we utilized several databases to evaluate NNMT expression and its link with prognosis across cancers. Additionally, we investigated the potential connections between NNMT expression and immunological infiltration levels, immune activation genes, immunesuppressive genes, MHC genes, chemokines genes, and chemokines receptor genes. In particular, we investigated the potential correlations between NNMT expression and mutation landscape, treatment sensitivity, microsatellite instability (MSI), tumor mutational burden (TMB), and DNA methylation in 33 cancer types. To further explore the bioactivity of NNMT in malignancies, co-expression analyses of immune-related and mismatch repair (MMR) genes with NNMT, protein-protein interact (PPI) network, and enrichment analysis were performed. Our findings indicate that NNMT can be employed as a predictive factor and plays a crucial role in tumor microsatellite instability (TMI) through influencing tumor-infiltrating immune cells, genetic alteration, TMB, and MSI. This research can shed light on the function of NNMT in tumor immunotherapy.

## Materials and methods

### Data collection

**FIGURE 1:**
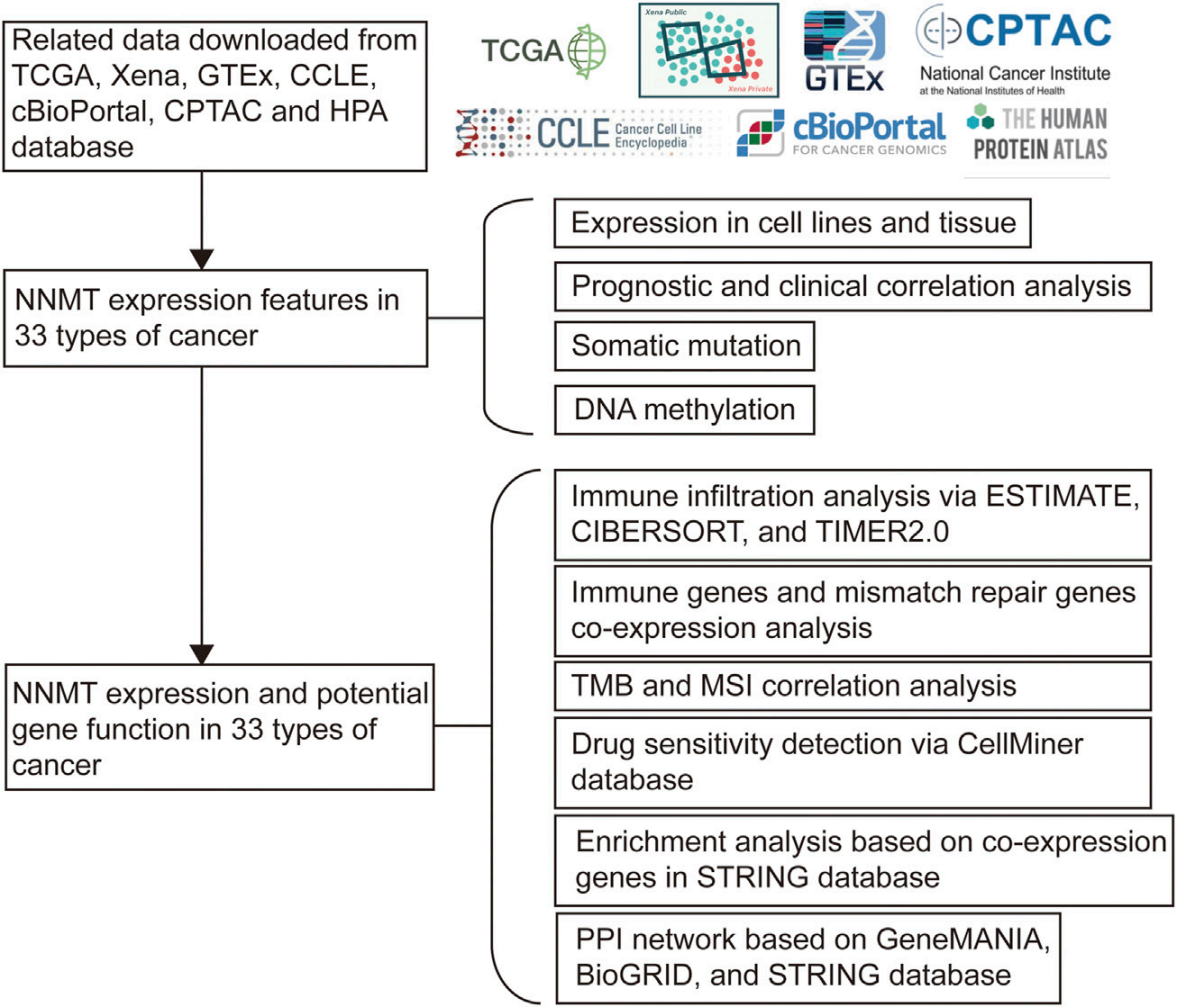
A flow chart of the study.

RNA sequencing, somatic mutation, and connected clinical data from The Cancer Genome Atlas (TCGA) and Broad Institute Cancer Cell Line Encyclopedia (CCLE), Genotype-Tissue Expression (GTEx) as well asClinical Proteomic Tumor Analysis Consortium (CPTAC), were extracted for analysis *via* their hub websites or *via* UCSC Xena (https://xena.ucsc.edu/). Applying “tidyverse” and “limma” package in the R (R studio version: 1.2.1335, R version: 3.6.1) (http://www.rproject.org/ https://www.rstudio.com/), the whole data set was filtered, omitting missing and replicated results, and transformed to log_2_ (TPM +1). Strawberry Perl (Version 5.32.0, http://strawberryperl. com/) was used to retrieve NNMT gene expression data from Xena data sets for subsequent analyses. A total of 11,069 samples were gathered from TCGA, 981 cell types from CCLE, and 5838normal tissues from GTEx (Figure 1, Supplementary Methods). TMB was calculated by measuring the frequency of point mutation events in repeated genetic sequences, while MSI was computed by calculating the number of mutations per 1,000,000 base pairs. The expression of NNMT was evaluated in 31 normal tissues, 21 tumor cell lines, and 33 tumors, and expression levels were compared between cancer samples and matching terms in 33 cancers. *p* < 0.05 considered differential expression between tumor and normal tissues. Using the “ggplot2” package, the collected data was analyzed.

### IHC (immunohistochemistry) staining

To investigate changes in NNMT expression together with protein expression, IHC photos of NNMT protein in normal tissues and seven cancerous and normal tissues, including glioblastoma (GBM), pancreatic adenocarcinoma (PAAD), stomach adenocarcinoma (STAD), colon adenocarcinoma (COAD), kidney renal clear cell carcinoma (KIRC), and liver hepatocellular carcinoma (LIHC), were downloaded and studied from the HPA (http://www.proteinatlas.org/).

### Cox evaluation of regression and survival analysis

The connection between the expression of NNMT and overall survival (OS), disease-free survival (DFS), and disease-free interval (DFI) was analyzed by Cox regression *via* coxph function in the survival package (rdocumentation.org/packages/survival). Furthermore, to study the survival curve, the Kaplan-Meier method was used to generate the survival curve based on the NNMT express group using the best separation splinting strategy in R. Using the log-rank test, the difference between curves was examined, and a *p*-value less than 0.05 was deemed significant.

### Analysis of the prognosis in relation to pathological stage or age groups

Clinical phenotypic data encompassing American Joint Committee on Cancer pathological stage and gender were collected for each sample obtained from TCGA, then integrated with the NNMT expression matrix to examine the correlation between NNMT levels and pathological stage or age groups. Patients were split into two groups based on a cutoff age of 65 years. The R package “ggplot2” was used to plot correlation analyses of clinical phenotypes.

### The correlation between nicotinamide n-methyltransferase expression and immune environment

The Estimation of Stromal and Immune Cells in Malignant Tumor Tissues Using Expression Data (ESTIMATE, version 1.0.13) algorithm was used to assess immune infiltration on behalf of stromal score and immune score. Furthermore, we conducted CIBERSORT, a method for analyzing the cell composition of tumor tissues from the gene expression profiles in 26 types of cancer except for cholangiocarcinoma (CHOL), diffuse large B-cell lymphoma (DLBC), lung adenocarcinoma (LUAD), mesothelioma (MESO), pheochromocytoma and paraganglioma (PCPG), testicular germ cell tumors (TGCT) and thymoma (THYM) due to results containing not available value. Using the R package “IOBR” and the deconvo CIBERSOR option, associations between NNMT level and each grade of immune cell infiltration in malignancy were investigated (*p* < 0.05 was deemed significant).

Tumor Immune Estimation Resource 2.0 (TIMER2.0) was a data program that computes immune cell infiltration scores for six principal immune cell types, specifically, CD8^+^ T cells, CD4^+^ T cells, macrophages, myeloid dendritic cells, neutrophils and B cells. In the “Immune” module, infiltration data were obtained and analyzed to see whether NNMT expression was associated with infiltration.

Moreover, a co-expression analysis was undertaken using the R packages “reshape2” and “RColorBrewer” to choose immune activation genes, chemokine genes, chemokines receptors genes, MHC genes, and immunesuppressive genes.

### Nicotinamide n-methyltransferase expression and drug response

CellMiner (https://discover.nci.nih.gov/cellminer/analysis.do) was a database designed for the cancer research community to test over 100,000 chemical compounds and natural products in 60 unique human cancer cell lines (NCI-60) utilized by the Developmental Therapeutics Program of the National Cancer Institute of the United States, Center for Cancer Research and National Institutes of Health since 1990 (Reinhold et al., 2012). CellMiner suggested a connection between NNMT expression and medication sensitivity.

### PPI network of nicotinamide n-methyltransferase

GeneMANIA (http://www.genomania.org) is an interactive database of protein-protein, protein-DNA, pathways, reactions, protein domains, and phenotypic screening profiles that provides gene function prediction and genes with associated functions (Warde-Farley et al., 2010). GeneMANIA was therefore employed to investigate NMT PPI in this study. Likewise, the NNMT PPI network was constructed using BioGRID 4.4 (https://thebiogrid.org/) “Network” module and “Concentric Circles” layout (Oughtred et al., 2021). In addition, the STRING 11.5 (https://string-db.org) was utilized to generate a Homo Sapiens NNMT co-expression network with the following primary parameters: 1) Meaning of network edges: evidence; 2) Sources of active interaction: co-expression; 3) Maximum number of interactors: 10; and 4) Minimum required interaction score: low confidence (0.150). In addition, pairwise gene correlation analysis was performed using the GEPIA2 “Correlation Analysis” module in all TCGA tumors.

### Nicotinamide n-methyltransferase-related gene enrichment analysis

To investigate NNMT protein function, the string database was utilized to identify co-expression proteins with the following parameters: the minimum required interaction score “Low confidence (0.150)", the meaning of network edges (“evidence”), the maximum number of interactors to display (“no more than 50 interactors” in the first shell), and active interaction sources (“Co-expression”). On the official GSEA website (https://www.gsea-msigdb.org/gsea/downloads.js), gene ontology (GO) and Kyoto Encyclopedia of Genes and Genomes (KEGG) gene sets were downloaded. The “clusterProfiler” (version 3.14.3) R package was used to perform GO and KEGG enrichment analysis utilizing the gene symbols of these 50 genes as input.

### Methylation profile and methylation related survival of Nicotinamide n-methyltransferase

DNA methylation is an essential aspect of NNMT activity, and as a key regulator of gene transcription, it has the potential to trigger cancer. Utilize HM450 methylation information from cBioPortal (www.cbioportal.org) and the proteome information from CPTAC (https://pdc.cancer.gov/pdc/). In each tumor, the relationship between NNMT expression and gene promoter methylation was studied. The Kaplan-Meier approach was utilized to determine the relationship between NNMT methylation and prognosis, including OS, DSS, PFI, and DFS.

## Results

### Transcriptional expression and protein expression of nicotinamide n-methyltransferase in diverse normal and cancer tissues

**FIGURE 2:**
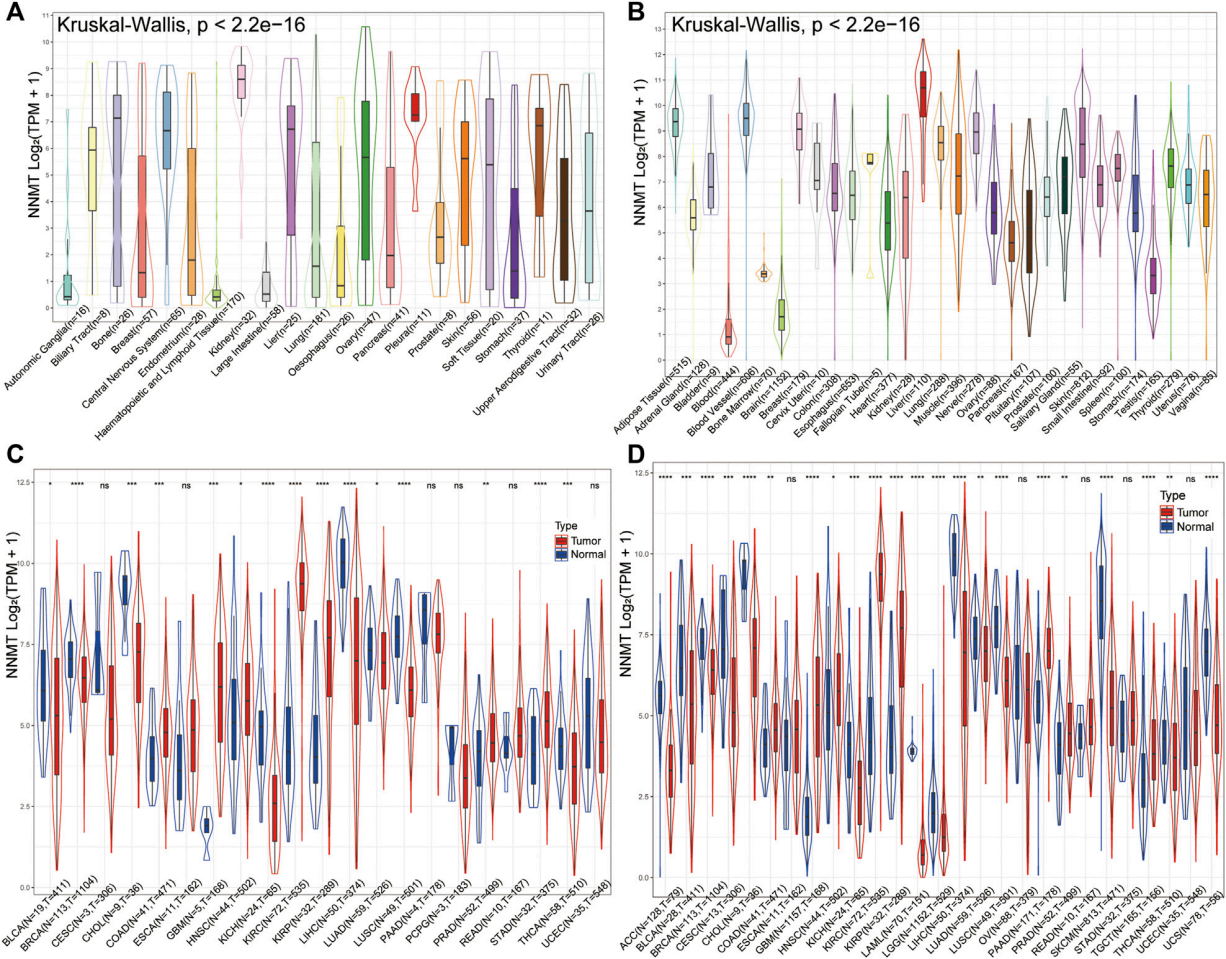
Differential expression of NNMT. **(A)** NNMT expression in multiple normal organs from GTEx database; **(B)** NNMT expression levels in cancer cell lines from CCLE database; **(C)** NNMT expression differences between tumor and normal samples for 21cancers from TCGA data alone; **(D)** NNMT expression differences between tumor and normal sample combined TCGA and GTEx for 27 cancers. (“ns” means “no significance”, *p < 0.05, **p < 0.01, ***p < 0.001, ****p < 0.0001).

The mRNA expression of NNMT were presented in different cancer cell lines but was highly discrete, with low levels in autonomic ganglia, haematopoietic and lymphoid tissue and large intestine cancer cells but the highest in kidney from the CCLE database (Figure 2A). The expression levels of NNMT were shown comparably in normal sample organs from healthy persons (Figure 2B), with a relatively low level in bladder, bone marrow and brain, using data from the GTExdatabase. However, a compare between relatively normal samples and tumors disclosed that NNMT were highly expressed in bladder urothelial carcinoma (BLCA, *p* < 0.05), breast invasive carcinoma (BRCA, *p* < 0.0001), cholangiocarcinoma (CHOL, *p* < 0.001), kidney chromophobe (KICH, *p* < 0.0001), LIHC (*p* < 0.0001), LUAD (*p* < 0.05), lung squamous cell carcinoma (LUSC, *p* < 0.0001) and thyroid carcinoma (THCA, *p* < 0.001) normal samples, and were highly expressed in COAD (*p* < 0.001), Glioblastoma (GBM, *p* < 0.001), head and neck squamous cell carcinoma (HNSC, *p* < 0.05), KIRC (*p* < 0.0001), kidney renal papillary cell carcinoma (KIRP, *p* < 0.0001), prostate adenocarcinoma (PRAD, *p* < 0.01), STAD (*p* < 0.0001) tumor samples *via* TCGA database alone (Figure 2C). Furthermore, normal samples of Adrenocortical Carcinoma (ACC), cervical squamous cell carcinoma and endocervical adenocarcinoma (CESC), acute myeloid leukemia (LAML), brain lower grade glioma (LGG), skin cutaneous melanoma (SKCM) and uterine carcinosarcoma (UCS) were newly found highly expressed NNMT (All *p* < 0.0001), and tumor samples of PAAD (*p* < 0.0001) was newly found highly expressed NNMT when the data from TCGA database and GTEx database were combined (Figure 2D).

**FIGURE 3:**
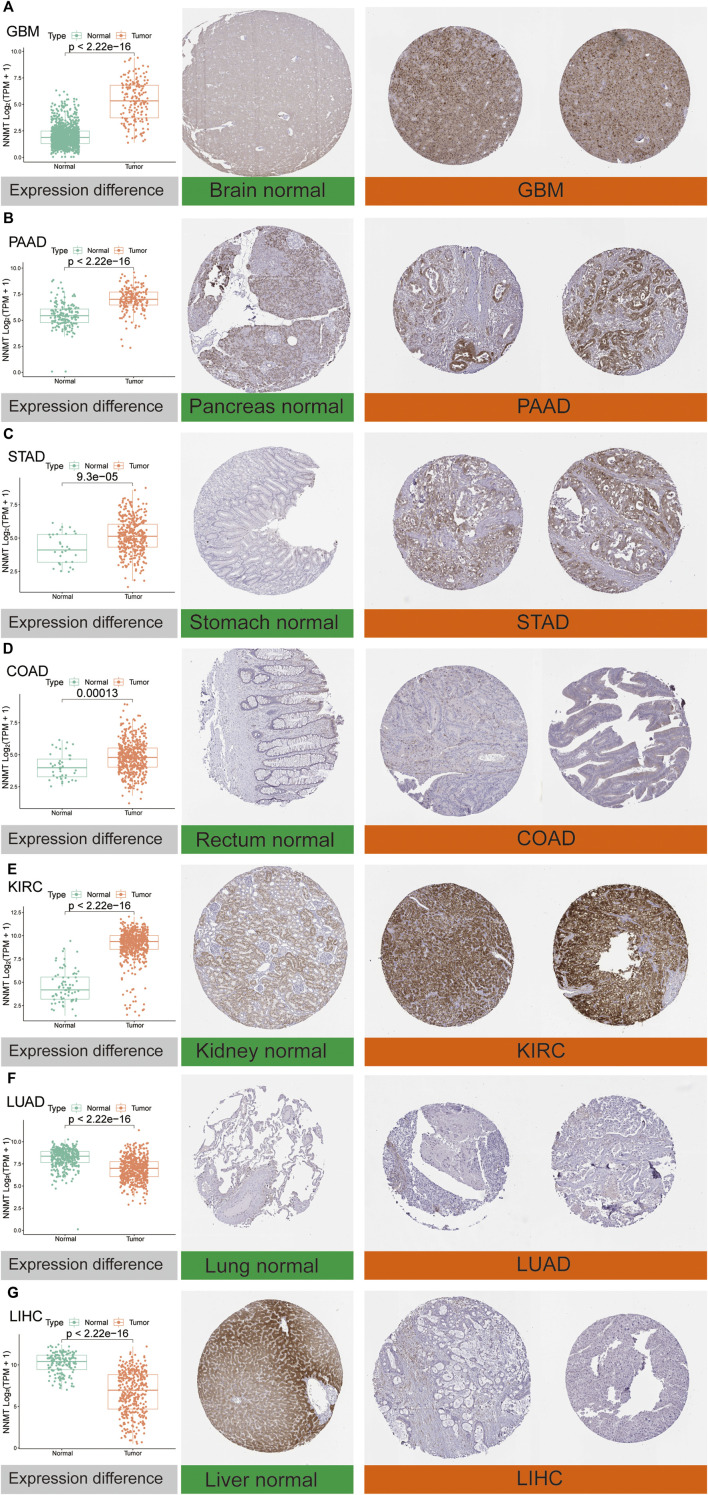
Comparison of NNMT expression at the mRNA level (left) and protein level (middle and right). Immunohistochemical images showed that NNMT protein was higher in GBM, PAAD, STAD, COAD, and KIRC, while it was lower in LUAD and LIHC compared with normal tissue. **(A)** Brain. **(B)** Pancreas. **(C)** Stomach. **(D)** Rectum. **(E)** Kidney. **(F)** Lung. **(G)** Liver.

Furthermore, to evaluate NNMT expression at the protein level, we explored IHC results of NNMT *via* the HPA database and compared mRNA expression data from TCGA. As was shown in Figures 3A–G, the analysis results from these two databases were consistent with one another. NNMT protein staining was relatively high in GBM, PAAD, STAD, COAD and KIRC cancer tissues compared with brain, pancreas, stomach, rectum and kidney normal tissues respectively. Conversely, normal lung and normal liver tissues had moderate NNMT staining, while LUAD and LUSC cancer tissues had weak NNMT staining respectively.

### The association between nicotinamide n-methyltransferase expression leveland prognosis of patients with cancer

**FIGURE 4:**
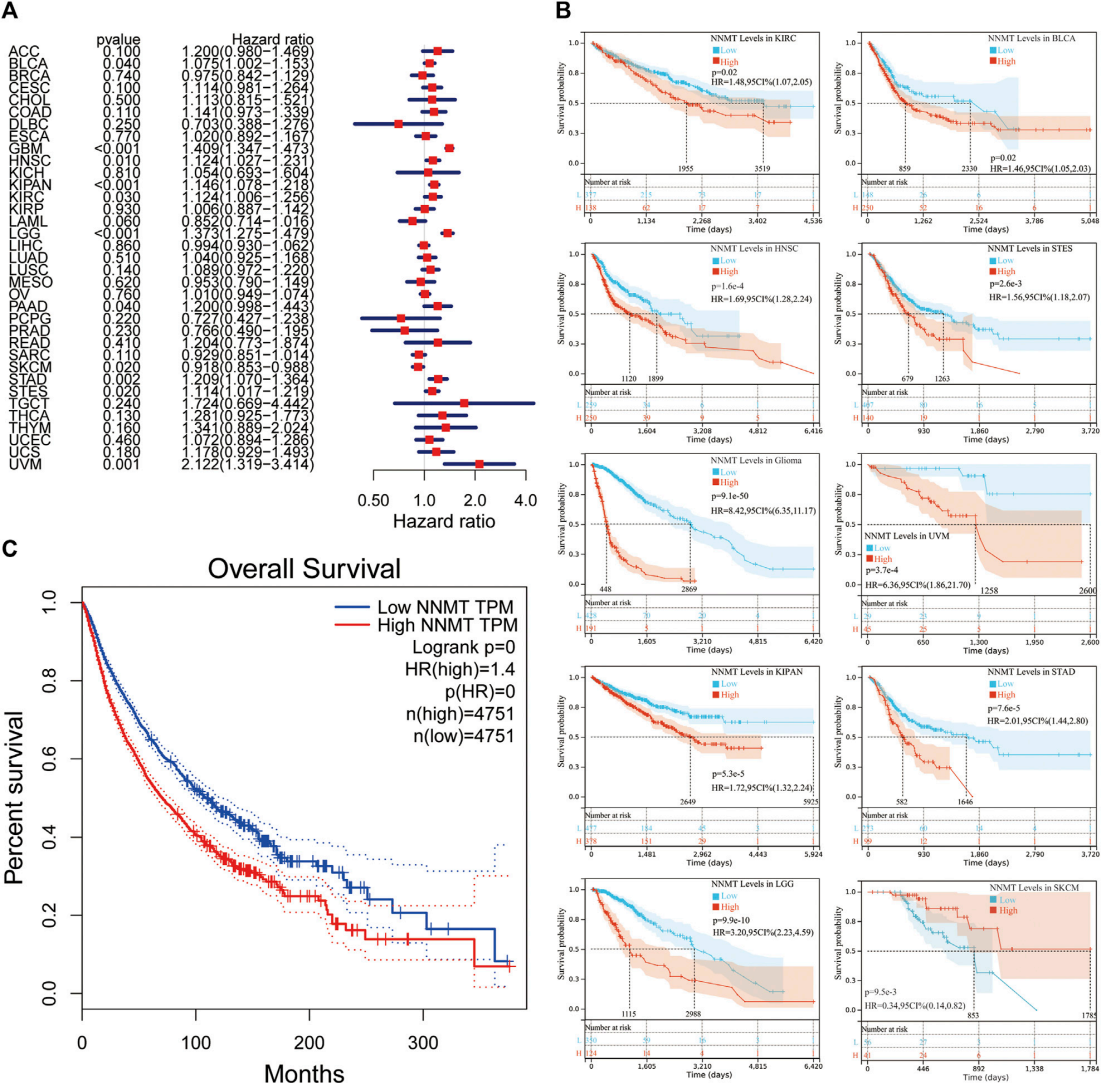
The relationship between NNMT expression and overall survival time in days (OS). **(A)** Cox regression evaluation of correlations between OS and 33 categories of tumors using a single variable. **(B)** Using data from the TCGA database, a high NNMT expression level was related with a shorter OS as compared to a low expression in 10 cancer types. **(C)** The Kaplan-Meier median expression value analysis between NNMT transcription and OS in 33 different cancers.

To examine the latent prognostic values of NNMT based on TCGA database, we explored the relationship between NNMT expression level and prognosis of patients with various tumors *via* single variate Cox regression analysis. The hazard ratios for NNMT were significant (*p* < 0.05) for HNSC, KIRC, LAML, LGG, PAAD, SKCM, STAD and uveal melanoma (UVM), among which NNMT had the highest risk effect in UVM (Figure 4A). Subsequently, the survival researches were conducted, which used patient data dissected for optimal cut-off level in several cancer types and indicated that survival variations in OS related cancer types were significant, indicating that patients with high NNMT expression accompanied low-quality outcomes (Figure 4B). Furthermore, a high NNMT expression level was linked with a low-quality OS through 33 cancer types shown in Figure 4C (HR = 1.4) compared to a low expression level using TCGA database.

**FIGURE 5:**
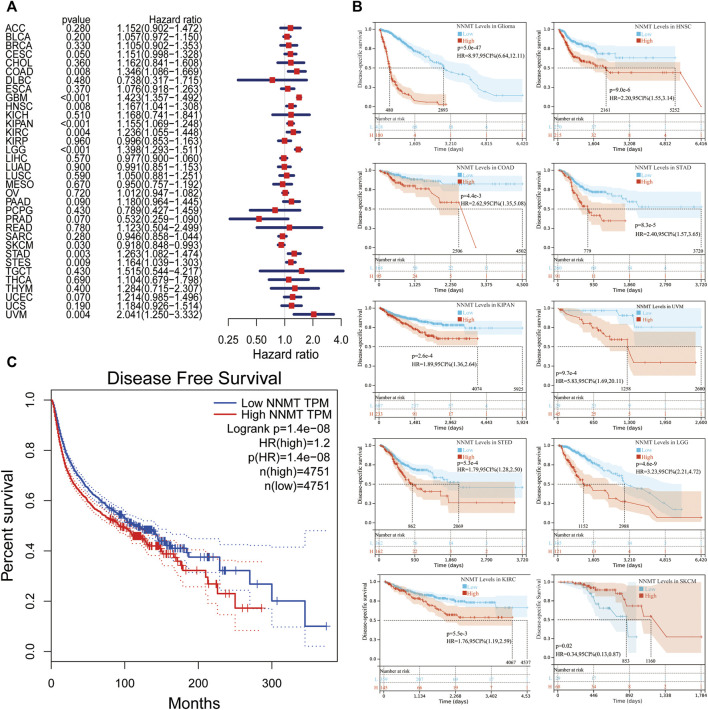
The correlation between disease-specific survival and NNMT expression in days (DSS). **(A)** Cox regression analysis of associations between DSS and 33 tumor types using a single variable. **(B)** A Kaplan-Meier analysis of the relationship between NNMT expression and DSS **(C)** The Kaplan-Meier median expression value analysis between NNMT transcription and disease-free survival (DFS) in 33 unique types of cancer.

**FIGURE 6:**
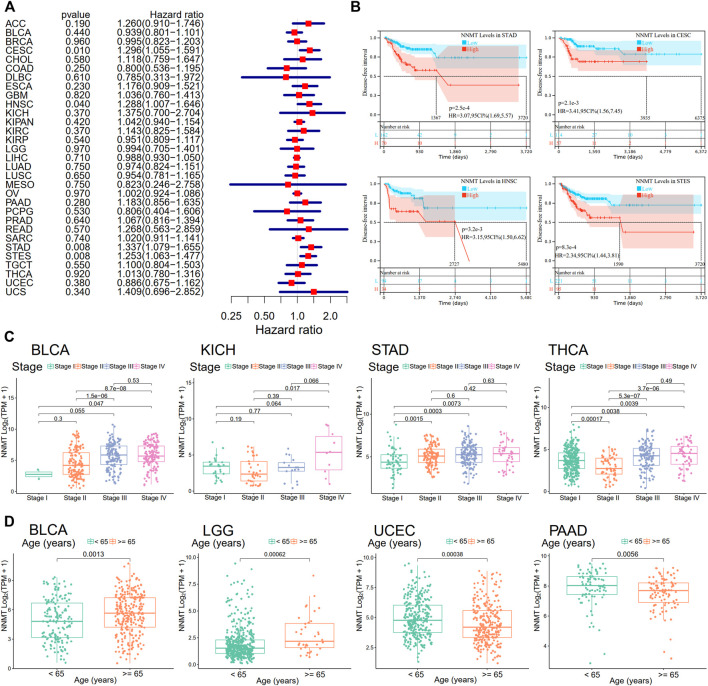
Relationship of NNMT expression with disease-free interval (DFI). **(A)** A forest plot shows the correlation between DFI and NNMT expression in 33 types of tumors. **(B)** A Kaplan-Meier analysis demonstrating the relationship between NNMT expression and DFI. The link between NNMT expression and pathological stages or age groups, including **(C)** BLCA, KICH, STAD, and THCA; and **(D)** BLCA, LGG, UCEC, and PAAD, applying the TCGA database.

Next, utilizing Cox regression, the correlation between NNMT expression and disease-free survival (DFS) and disease-specific survival (DSS) also were analyzed. OS correlation analysis and Cox regression analysis produced similar results. DSS significant effects (*p* < 0.05) were identified for BLCA, GBM, HNSC, pan-kidney cohort (KIPAN), KIRC, LGG, PAAD, SKCM, STAD, stomach, and esophageal carcinoma (STES), and UVM. In additionally, LAML cannot calculate a hazard ratio for NNMT due to a lack of relevant data (Figure 5A). In the following survival analysis, cancer types with high NNMT expression again had a poorer quality of life compared to those with low expression (Figure 5B). Similarly, a high level of NNMT expression was thought to be associated with a worse DFS in the TCGA cancer types as seen in Figure 5B (HR = 1.2, Figure 5C). Next, we performed a disease-free interval (DFI) analysis to NNMT levels using cox regression, but only found significant impacts (*p* < 0.05) for CESC, HNNSC, STAD, and STES (Figure 6A). Again, in the subsequent survival study, cancer types with high NNMT expression showed inferior DFI compared to those with low expression (Figure 6B).

### Nicotinamide n-methyltransferase expression was substantially related with advanced stages of cancer and older age groups

We examined the relationship between NNMT expression and the pathological stage of malignancies using the ggplot2 package. Figure 6C demonstrated that BLCA, KICH, STAD, and THCA were all substantially linked with tumor stage (*p* < 0.05). In addition, age groups were correlated with NNMT expression for BLCA, LGG, uterine corpus endometrial carcinoma (UCEC), and PAAD (Figure 6D, *p* < 0.05).

### Correlation between cancer nicotinamide n-methyltransferase expression and immunology penetration level

**FIGURE 7:**
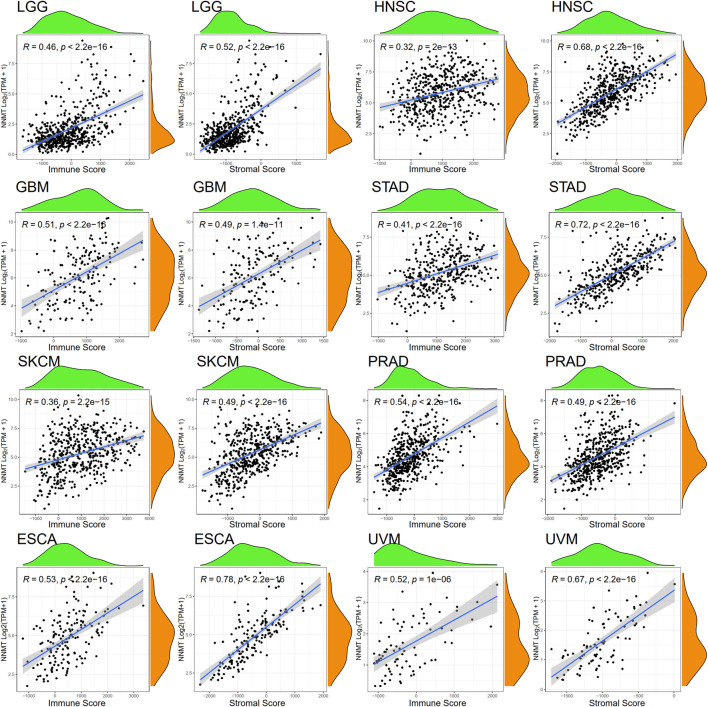
LGG, HNSC, GBM, STAD, SKCM, PRAD, ESCA, and UVM had the strongest significant correlation coefficients between the degree of tumor microenvironment and NNMT expression by Spearman’s rank correlation (immune scores and stromal scores, respectively).

By altering epigenetic alterations and acting in several signaling pathways, NNMT-mediated depletion of NAM and S-adenosyl methionine (SAM) leads to metabolic and epigenetic reprogramming in cancers. To evaluate if this route has an influence on the immunological environment of tumors, we analyzed the relationship between NNMT expression and the degree of immune cell infiltration in 33 cancers. From the TCGA database, 31 of 33 tumor types were significantly (*p* < 0.05) positively correlated with NNMT expression except for MESO and LAML (Stromal Score), and 29 of 33 tumor types were significantly (*p* < 0.05) positively correlated with NNMT expression except for LAML (Immune Score), with THYM, diffuse large B-cell lymphoma (DLBC), and LUAD not significantly correlated (Supplementary Table S1; Supplementary Figure S1). LGG, HNSC, GBM, STAD, SKCM, PRAD, esophageal carcinoma (ESCA), and UVM were selected to demonstrate the association between immune infiltration and NNMT expression (Figure 7).

**FIGURE 8:**
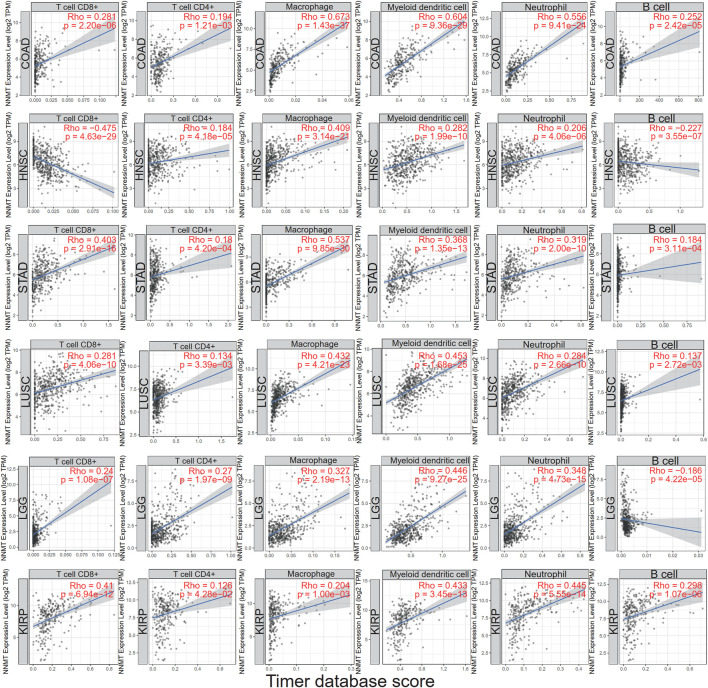
Using the infiltration scores of six immune cell types (CD8^+^ T cell, CD4^+^ T cell, macrophage, myeloid dendritic cell, neutrophil, and B cell) available in the TIMER2.0 database, a correlation was shown between NNMT expression and the degree of immune cell infiltration in different cancers.

Using the infiltration scores of six immune cell types (CD8^+^ T cell, CD4^+^ T cell, macrophage, myeloid dendritic cell, neutrophil and B cell) accessible in the Tumor Immune Estimation Resource (TIMER2.0) database and collected from TCGA, we discovered a substantial relationship in different malignancies. COAD, LUSC, and LGG were the three most common tumor types. The accompanying linear regression graphs from COAD, STAD, LUSC, and KIRP revealed a correlation between high NNMT expression and enhanced immune cell infiltration. In contrast, there was a negative association between NNMT expression and CD8^+^ T cell and B cell in HNSC and B cell in LGG. Dendritic cells exhibited the highest significant coefficients among all cell types in each of the six malignancies. In addition, macrophages were expected to have the highest STAD coefficients (Figure 8). Consequently, the results demonstrated that NNMT expression was tightly linked with the degree of immune infiltration in the majority of malignancies.

Using CIBERSORT, we also assessed the link between NNMT expression levels and the invasion of 22 immune-related cells. Our findings suggest that immune cell infiltration levels were substantially correlated with NNMT expression inside the vast majority of cancer types (Supplementary Table S2). Six tumors, including BLCA (*n* = 13), COAD (*n* = 10), KIRC (*n* = 10), LIHC (*n* = 10), ovarian serous cystadenocarcinoma (OV) (*n* = 14), and THCA (*n* = 17), with the strongest correlation between NNMT expression and degree of immune cell infiltration were examined further (Table 1). In the six tumors examined, NNMT expression levels were linked positively with invading naïve B cells and M1 macrophages but negatively correlated with naïve CD4 T cells, resting NK cells, and Eosinophils. In additionally, there was a link among NNMT expression levels and numerous subgroups of infiltrating macrophages. NNMT expression was significantly connected with levels of infiltrating M0 macrophages in BLCA, OV, and COAD, and with levels of infiltrating M1 macrophages in the six tumors analyzed. Similarly, except for KIRC and THCA, there were positive relationships between NNMT expression and levels of invading M2 macrophages in these six tumors.

**TABLE 1:** Relationship between NNMT expression and immune cell infiltration in different cancers.

Cell type	BLCA (Cor/P-value)	COAD (Cor/P-value)	KIRC (Cor/P-value)	LIHC (Cor/P-value)	OV (Cor/P-value)	THCA (Cor/P-value)
Naïve B cells	0.20/***	0.03	0.01	0.05	0.08	0.21/***
Memory B cells	-0.35/***	-0.03	-0.06	-0.12/*	-0.23/***	0.13/**
Plasma cells	-0.10	-0.41/***	0.05	0.04	-0.01	0.12/**
CD8 T cells	0.03	-0.10	0.13/**	0.13/*	0.00	-0.19/***
Naïve CD4 T cells	-0.33/***	-0.09	-0.08	-0.16/**	-0.19/***	-0.09/*
Resting CD4 memory T cells	0.12/*	-0.14/*	-0.02	0.14/**	0.35/***	0.12/**
Activated CD4 memory T cells	0.23/***	-0.10	0.01	0.05	0.11/*	0.10/*
Follicular T helper cells	-0.32/***	-0.18/**	0.10/*	-0.09	-0.28/***	-0.01
Regulatory T cells	-0.19/***	0.01	0.10/*	0.16/**	-0.01	0.17/***
Gamma delta T cells	-0.07	-0.07	0.06	0.06	-0.03	0.10/*
Resting NK cells	-0.13/**	-0.07	-0.14/**	-0.15/**	-0.12/*	-0.32/***
Activated NK cells	0.002	-0.01	-0.06	-0.01	0.03	-0.07
Monocytes	-0.09	0.02	-0.15/***	-0.12/*	-0.15/**	-0.19/***
M0 Macrophages	0.16/***	0.15/*	-0.03	-0.02	-0.17/***	0.03
M1 Macrophages	0.31/***	0.23/***	0.12/**	0.03	0.21/***	0.27/***
M2 Macrophages	0.37/***	0.28/***	-0.01	0.06	0.14/**	-0.09/*
Resting dendritic cells	-0.08	-0.05	-0.06	0.03	0.08	0.40/***
Activated dendritic cells	-0.30/***	-0.17/**	-0.10/*	-0.04	0.01	0.09/*
Resting mast cells	-0.03	0.18/**	-0.26/***	-0.30/***	-0.24/***	-0.31/***
Activated mast cells	-0.02	-0.12/*	0.07	0.06	0.16/***	-0.08
Eosinophils	-0.19/**	-0.06	-0.26/***	-0.13/*	-0.18/***	-0.25/***
Neutrophils	0.10/*	0.14/*	0.12/**	0.11/*	0.26/***	-0.01

BLCA, bladder urothelial carcinoma; COAD, colon adenocarcinoma; KIRC, kidney renal clear cell carcinoma; LIHC, liver hepatocellular carcinoma; OV, ovarian serous cystadenocarcinoma; THCA, Thyroid carcinoma. *p < *0.05,* **p < *0.01, and* ***p < *0.001*.

### Correlation between NNMT expression and immune gene expression in certain cancers implicates nicotinamide n-methyltransferase in the tumor immune response

**FIGURE 9:**
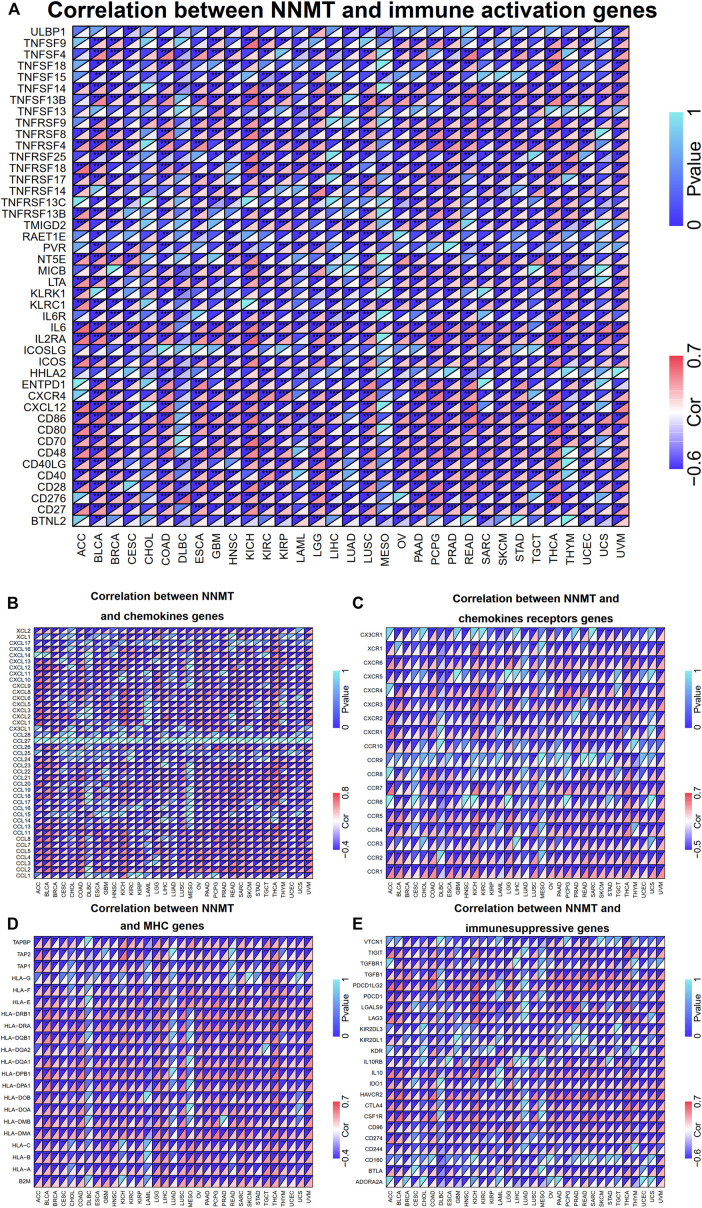
Co-expression of NNMT with genes involved in immunity. The triangle in the upper left corner indicated the P-value, whereas the triangle in the lower right corner represented the correlation coefficient. **(A)** The expression of NNMT was positively associated with immune activation markers such as CD86, CD27, and IL-6. Expression of NNMT was positively correlated with the majority of chemokines genes **(B)** and a portion of chemokines receptors, including CCR1, CCR2, and CXCR6 **(C)**. NNMT expression was favorably associated with the majority of MHC genes **(D)**, as well as immunesuppressive genes including HAVCR2, CTLA4, and CSF1R **(E)**. (*p < 0.05, **p < 0.01, ***p < 0.001).

Multiple genes, comprising chemokine, chemokine receptor, MHC molecule, immunoinhibitor, and immunostimulator, were closely associated with and recognized as immune response genes. Applying TCGA databases, we were capable of assessing whether there is a relationship between NNMT expression and the expression of such immune genes. In various types of cancer, findings between NNMT and immunostimulator gene expression showed a high connection (*p* < 0.05), including TNFSF13B, TNFRSF8, TNFSF14, IL6, IL2RA, CD80, CD27, and CD86 (Figure 9A). Regarding chemokine genes, NNMT expression is significantly (*p* < 0.05) positively correlated with XCL2, CCL13, CCL11, CCL5, and CCL2. Similar to NNMT, receptor genes like CXCR6, CXCR4, CCR7, CCR5, CCR2, and CCR1 were similarly substantially (*p* < 0.05) positively associated with receptor genes (Figures 9B,C). As for MHC-encoding genes, NNMT was significantly (*p* < 0.05) associated with HL-A class genes include HLA-F, HLA-DRA, HLA-DMA, and HLA-A (Figure 9D). NNMT was significantly (*p* < 0.05) positively linked with the immunoinhibitor genes TGFB1, IL10, HAVCR2, CTLA, and CD96 (Figure 9E). In addition, BLCA, LUSC, KICH, and UVM all displayed considerable co-expression of NNMT with key immune checkpoint genes. The findings suggested that NNMT could be involved in the regulation of the tumor immune response by altering the activity of immune genes.

Our study confirmed that the expression of NNMT was tightly associated with the biological processes of immune cells and was co-expressed with genes encoding MHC, immunological activation, immunosuppression, chemokine, and chemokine receptor genes in the vast majority of malignancies. Measuring the level of NNMT expression could be a valuable method for determining the efficacy of immunotherapy.

### The genetic alteration landscape of nicotinamide n-methyltransferase and mutation landscape across different tumors

**FIGURE 10:**
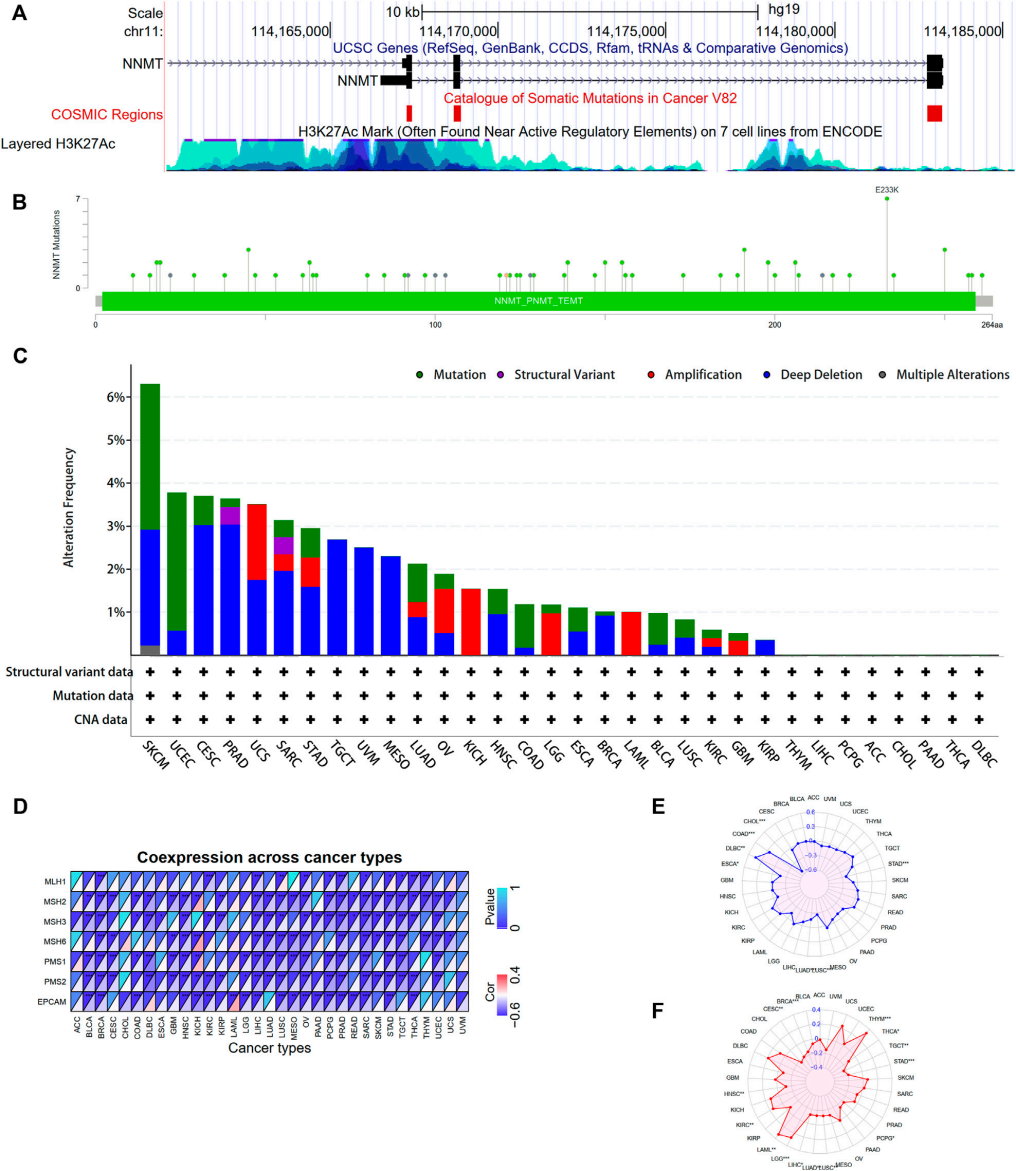
NNMT gene mutation analysis and associations between NNMT expression and mismatch repair (MMR), microsatellite instability (MSI) and tumor mutational burden (TMB). **(A)** NNMT gene mutation sites and epigenetic modifications were visualized using the UCSC genome browser. NNMT genetic alteration in various tumor types of TCGA. The alteration frequency with NNMT genetic alteration type **(B)** and NNMT mutation site **(C)** were generated by cBioPortal. **(D)** NNMT expression was significantly and highly connected with MMR gene expression in the majority of cancer types (except ACC CHOL and UVM); MSH2 MSH3 PMS1 and PMS2 were negatively correlated with NNMT in the majority of cancer types. **(E)** The expression of NNMT was inversely connected with MSI in CHOL, ESCA, LUAD, LUSC, and STAD, but positively correlated with COAD and DLBC. **(F)** In KIRC, LAML, LGG, and THYM, NNMT expression was positively connected with TMB, but it was negatively correlated in BRCA, CESC, HNSC, LIHC, LUAD, LUSC, PCPG, STAD, TGCT, and THCA. (*p < 0.05, **p < 0.01, ***p < 0.001).

To investigate NNMT mutation features and the relationship of other mutation genes grouped by NNMT expression, we conducted mutation analysis across various tumors. The result shows that somatic mutation sites existed in NNMT exon region and H3K27Ac levels were high around regulatory elements sites of NNMT (Figure 10A). A significant amount of acetylation is present in the regulatory region of the NNMT gene, indicating a high level of transcriptional activity. As shown in Figures 10A,B total of 75 NNMT mutations, including 63 missense mutations, four nonsense mutations, three frame-shift deletions, one frame-shift insertion and three fusion mutations, were detected in TCGA tumor samples (Supplementary Table S3, 4). The residue 233 of the protein encoded by NNMT had seven mutations, making it the most frequently mutated region in the NNMT protein (Figure 10B). Besides, we found that SKCM tumor samples had the highest NNMT genetic alteration frequency (>6%). For all malignancies, deep deletion was the most prevalent mutation type, and the only type for TGCT, UVM, and MESO (Figure 10C; Supplementary Table S4). In KICH, only amplification mutation was detected and no mutations were detected in THYM, LIHC, PCPG, ACC, CHOL, PAAD, THCA and DLBC (Figure 10C).

### NNMT expression level correlations with mismatch repair genes, tumor microsatellite instability and tumor mutation burden

Subsequently, we studied the relationships between the expression of MMR genes, which including MLH1, MSH2, MSH3, MSH6, PMS1, PMS2, and EPCAM. The correlations between NNMT expression levels and those of distinct MMR genes are depicted in Figure 10D. We discovered a negative connection between NNMT expression and MMR gene expression in the majority of malignancies, excluding ACC, UVM, and CHOL.Furthermore, we investigated whether there were correlations between NNMT expression levels and MSI and TMB, which both have essential connections with immune checkpoint inhibitor sensitivity. NNMT expression was related with MSI in seven additional tumor types, including colorectal cancer, lung cancer, stomach cancer, and lymphadenoma (Figure 10E). NNMT expression was found to be associated with TMB in 14 types of tumors, including breast cancer, colorectal cancer, lung cancer, glioma, and kidney cancer (Figure 10F). And the mutations in the top 20 most frequently occurring genes in 14 TCGA cancers grouped by NNMT levels, respectively, are shown in Supplementary Figure S2. In the low NNMT level group, TP53 mutations were more prevalent than in the high expression group, with the exception of sarcoma (SARC); mutation events were more common in the high NNMT level group for LGG, SARC, BLCA, KIRP, UCEC, COAD, and ESCA. The mutation frequency of NNMT in all malignancies was modest, but the expression of NNMT can greatly influence the mutation of some key genes, such as TP53, EGFR, and CDKN2A. It may be because the expression of NNMT influences the methylation level of oncogenes and tumor suppressor genes.

### Nicotinamide n-methyltransferase and drug response

**FIGURE 11:**
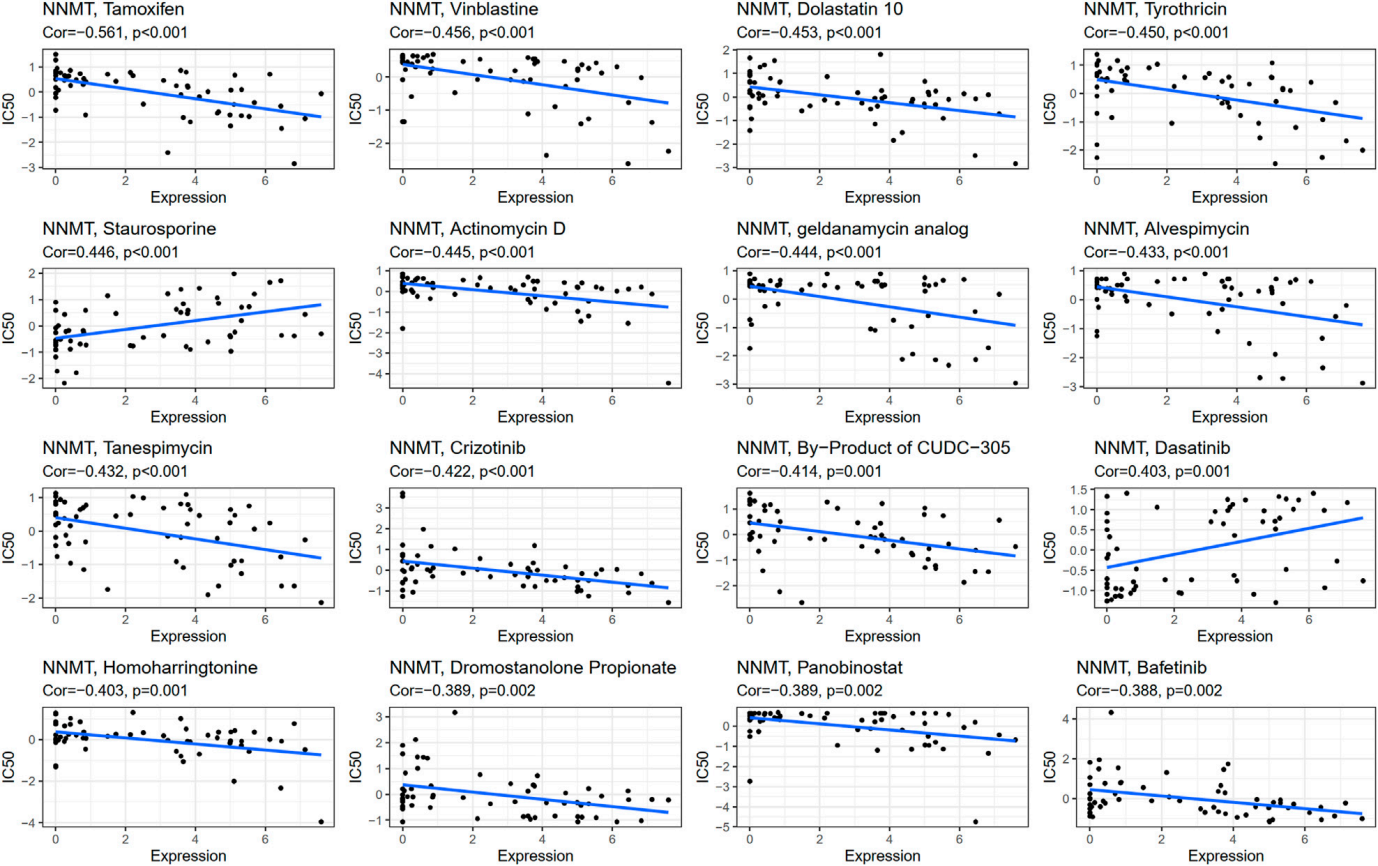
A depiction of the association between NNMT expression and predicted FDA drug reaction.

NNMT expression was negatively connected with drug response in patients treated with Tamoxifen, Vinblastine, Dolastatin 10, Tyrothricin, Actinomycin D, Geldanamycin Analog, Alvespimycin, Tanespimycin, Crizotinib, CUDC−305, Homoharringtonine, Dromostanolone Propionate, Panobinostat, and Bafetinib. Additionally, there is a positive connection between NNMT expression and the anticancer drug Staurosporine and Dasatinib. An illustration of the relationship between NNMT expression and expected medication response can be found in Figure 11; Supplementary Table S5.

### PPI network of nicotinamide n-methyltransferase and enrichment analysis for cancer

**FIGURE 12:**
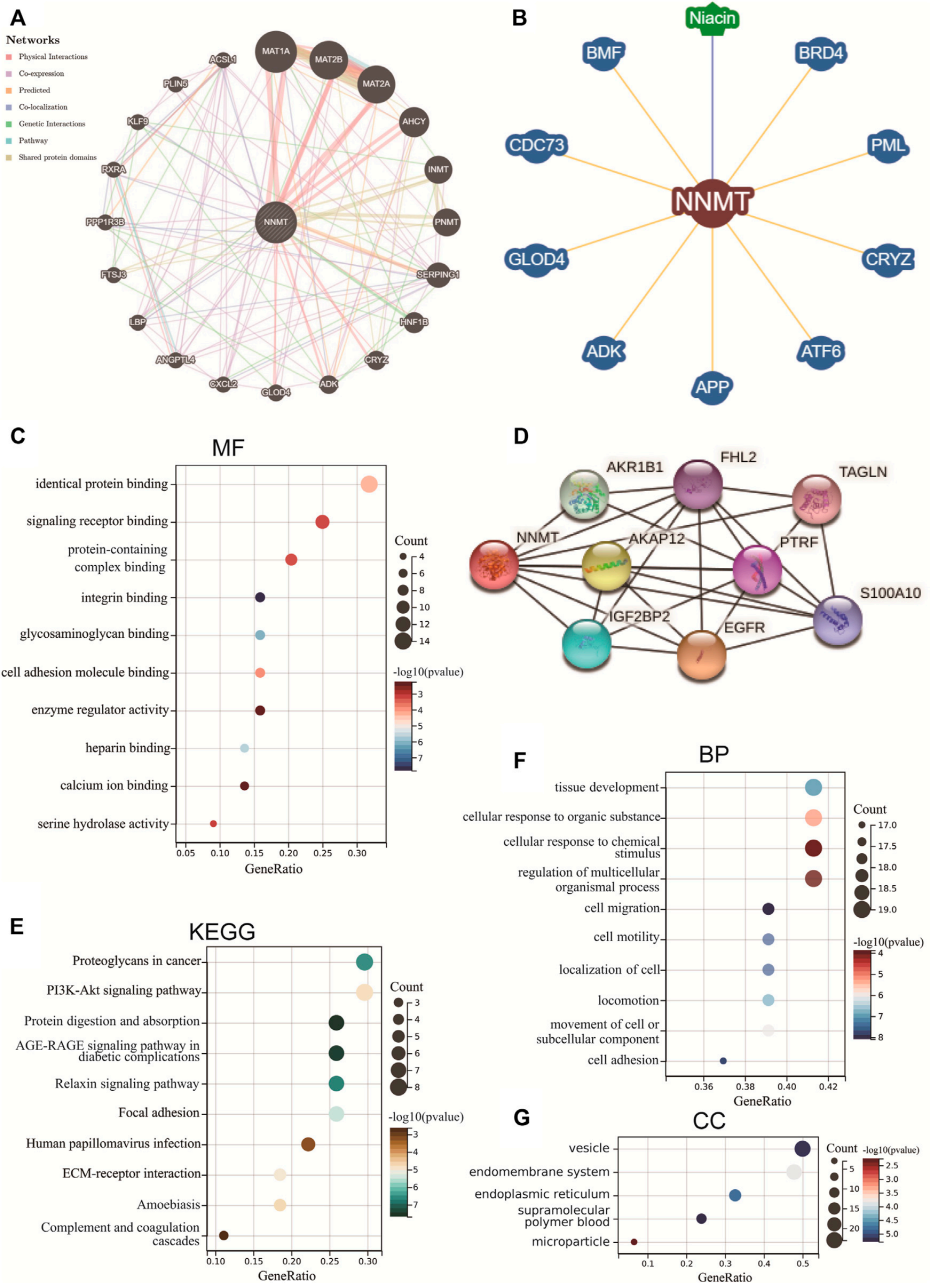
NNMT-related protein-protein interact (PPI) networks and gene enrichment analysis. PPI networks for NNMT using the GeneMANIA database **(A)**, BioGRID database **(B)** and STRING database **(D)**. **(C)** Molecular function (MF), **(E)** Kyoto Encyclopedia of Genes and Genomes (KEGG), **(F)** biological process (BP) and **(G)** cellular components (CC) analysis of the top 50 genes co-expressed with NNMT obtained by the STRING.

To investigate the functional mechanism of NNMT in carcinogenesis, we used STRING to extract the top 50 genes with expression patterns similar to NNMT for enrichment analysis (Supplementary Table S6). Next, we utilized the GeneMANIA, BioGRID and STRING online databases to create a PPI network for NNMT, which is displayed in Figures 12A,B,D, to investigate the probable processes by which NNMT played a role in cancer carcinogenesis. NNMT demonstrated significant physical interactions with MAT1A, MAT2B, MAT2A, AHCY and INMT, as illustrated in the Figure 12A. According to the BioGRID4.3 database, NNMT physically interacts with CDC73, ADK, and ATF6 (Figure 12B), which have well-characterized functions in the mitogenesis, chemoresistance, and tumorigenesis. And the Pearson correlation coefficients between NNMT and CRYZ, and SERPING1 and ANGPTL4 across all TCGA tumor samples were 0.4, 0.43, and 0.62, respectively (Supplementary Figure S3).

Following that, co-expressed genes with NNMT were showed *via* the STRING tool and indicated that NNMT was related to EGFR, AKR1B1, IGF2BP2, AKAP12, FHL2, PTRF, TAGLN and S100A10 (Figure 12D). These findings prompted us to wonder whether NNMT plays a role in these biological processes by interacting with key proteins involved in cell function. Furthermore, the enriched molecular functions (MF) were linked to Identical protein binding/Signaling receptor binding/Protein-containing complex binding/Integrin binding/Glycosaminoglycan binding/Cell adhesion molecule binding/Enzyme regulator activity/Heparin binding/Calcium ion binding/Serine hydrolase activity (Figure 12C, Supplementary Table S6). According to the KEGG analysis, the far more significantly enriched pathways engaged Proteoglycans in cancer, PI3K-Akt signaling pathway, Protein digestion and absorption, AGE-RAGE signaling pathway in diabetic complications, Relaxin signaling pathway, Focal adhesion, Human papillomavirus infection, ECM-receptor interaction, Amoebiasis, and Complement and coagulation cascades (Figure 12E, Supplementary Table S8). The biological processes (BP) enriched in this dataset were primarily those related to Tissue development/Cellular response to organic substance/Cellular response to chemical stimulus/Regulation of multicellular organismal process/Cell migration/Cell motility/Localization of cell/Locomotion/Movement of cell or subcellular component/cell adhesion, while the cellular components (CC) enriched were primarily those related to Vesicle/Endomembrane system/Endoplasmic reticulum/supramolecular polymer/blood microparticle (Figure 12F; Supplementary Table S9; Figure 12G; Supplementary Table S10).

### The negative correlation between nicotinamide n-methyltransferase methylation profile and mRNA

**FIGURE 13:**
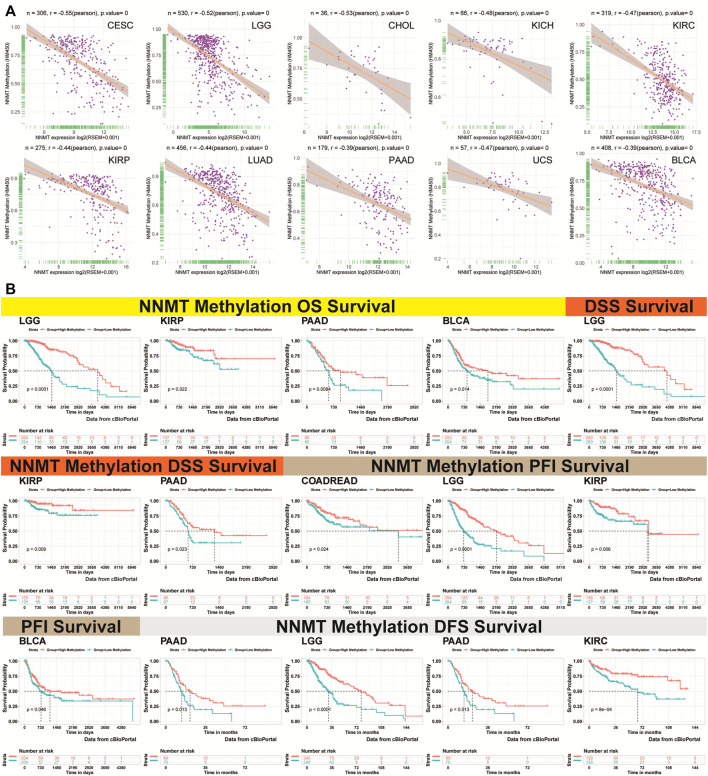
Association between NNMT expression and methylation of gene promoters. **(A)** Correlation between NNMT expression and gene promoter methylation in the 10 most prevalent malignancies, namely CESC, LGG, CHOL, KICH, KIRC, KIRP, LUAD, PAAD, UCS, and UVM. **(B)** Correlation between NNMT methylation OS in LGG, KIRP, PAAD, and BLCA; DSS in LGG, KIRP, and PAAD; PFI in colon adenocarcinoma/rectum adenocarcinoma esophageal carcinoma (COADREAD), LGG, KIRP, and BLCA; and DFS in PAAD, LGG, KIRP, and COADREAD.

The topography of NNMT methylation in pan-cancer was also studied. We calculated the Pearson correlation between NNMT promoter methylation and mRNA levels *via* cBioPortal data and found significance in 28 types cancer (Supplementary Figure S3). Figure 13A depicted the ten strongest negative relationships (CESE, LGG, CHOL, KICH, KIRC, KIRP, LUAD, PAAD, UCS, and BLCA). Exceptions include ACC, SARC, PCPG, DLBC, UVM, and TGCT. In the majority of cancer types, NNMT methylation was found to be closely associated with NNMT mRNA expression.

### The nicotinamide n-methyltransferase promoter methylation and prognosis analysis

We analyzed the link between NNMT promoter methylation and prognosis using Kaplan-Meier survival analysis for four indicators of OS, DSS, PFI, and DFS. In general, the poorer the clinical prognosis, the greater the NNMT promoter methylation level, and this trend is particularly obvious in LGG, KIRC, KIRP, and PAAD (Figure 13B). For glioma, NNMT low methylation of cg16208682, cg11771151, and cg10974001 indicated poor prognosis (Supplementary Figure S4). In protein level, NNMT showed same trend in glioma, KIRC, UCEC, LUAD and HNSC based on CPTAC database (Supplementary Figure S4; Supplementary Table 11).

## Discussion

In phase II methylation metabolism, NNMT modulates substrate activity by transferring the methyl group from SAM to the substrate. In cancer cells, the production of methylation sink decreases histone and DNA methylation (such as H3K27 and H3K4), modifies gene mutation and expression levels, and may result in an uncontrolled metabolic state (Ulanovskaya et al., 2013; Eckert et al., 2019). According to previous studies, NNMT was abundant in the cytoplasm and overexpressed in a variety of human malignant tumors, including lung cancer, glioblastoma, gastric cancer, pancreatic cancer, and colorectal cancer, suggesting that NNMT may be a co-carcinogenic factor in malignancies (Sartini et al., 2015; Chen et al., 2016; Xie et al., 2016; Xu et al., 2016; Palanichamy et al., 2017). Reportedly, NNMT was up-regulated in the livers of tumor-bearing mice and was implicated in the down-regulation of the urea cycle and uracil production, which may be involved in the process of protein breakdown and amino acid buildup in cancer cells that promotes tumor cell proliferation (Mizuno et al., 2022). In cancer-associated fibroblast cells, NNMT induced collagen contractility and globally regulated thousands of genes, including pro-tumorigenic cytokines, to enhance EMT and accelerate tumor growth (Eckert et al., 2019). One possible mechanism is that the expression of NNMT induces the methylation of genomic DNA, which significantly changes the methylation status of the gene promoter (Xu et al., 2005). Genes involved in collagen production and myosin-driven contraction are up-regulated in the tumor microenvironment and promote the growth of tumor cells (Orimo et al., 2005; Kalluri, 2016).

Our research revealed that NNMT expression was significantly variable in 24 types of tumors and paracancerous tissues, and up-regulated in nine types of malignancies, which was consistent with the immunohistochemical analysis of protein levels. The results of PAAD (Xu et al., 2016), STAD (Chen et al., 2016), KIRC (Reustle et al., 2022), GBM (Palanichamy et al., 2017), HNSC (Togni et al., 2021) and COAD (Ogawa et al., 2022) were similar to previous studies. However, the results suggested that there was a relatively high level of NNMT expression in the normal tissues of 15 distinct types of tumors, which may be a result of the vast variation in metabolic status between tumor types (Park et al., 2020; Hanahan, 2022). Deng et al. reported in a recent meta-analysis that increased NNMT expression was significantly associated with shorter OS and DFS, more advanced clinical stages, inferior tumor differentiation, earlier lymph node metastasis, and earlier distant metastasis (Dang and Wang, 2022). This was identical to our results that expression of NNMT was lower in younger individuals with BLCA and LGG, whereas expression of NNMT was lower in older patients with UCEC and PAAD. Also, our investigation demonstrated that NNMT expression was connected with the pathological stage in BLCA, KICH, STAD, and THCA, and was especially distinct from stages I and III. Serra Akar et al. observed that NNMT expression can be used as a prognostic factor for lymph node metastasis and staging in cervical squamous cell carcinoma with different clinical stages (Akar et al., 2020). However, in melanoma, elevated NNMT expression was related to a better prognosis, which has not been previously documented and may need future investigation.

TME provides a favorable environment for cancer cell expansion. Tumors survive through immune metabolic reprogramming and immune cell changes that occur in the TME, such as activating immune and stromal cells, such as macrophages, neutrophils, T cells, B cells, and fibroblasts, and initiating metabolic reprogramming, such as enhancing the “Warburg effect”, to promote tumorigenesis and development or produce an environment beneficial to tumor growth by competing for energy-limited resources such as oxygen and glucose, necessary and non-necessary nitrogen (Zhao et al., 2022). DNA methylation is intimately linked with TME because hypermethylation in the promoter region of tumor suppressor genes, such as TGF-β, SHP-1, and IFN-γ, frequently results in silencing of these genes (Zhang et al., 2017). Our results illustrated that NNMT was important for cancer immunity. According to ESTIMATE scores, there were favorable associations between NNMT expression and stromal and immune cell composition in the TME of 31 malignancies, and therefore a strong relationship with naïve B cells, M1 macrophages, naïve CD4 T cells, and resting NK cells. Tumor-infiltrating immune cells exert a significant influence on the formation and progression of tumors, and can inhibit or promote tumor occurrence and progression (Zhang and Zhang, 2020).

MSI is an important element in the occurrence and growth of cancers *via* the mechanism of oncogene mutation, and it assisted immunotherapeutic response. High microsatellite instability is an important prognostic factor for colorectal, gastric, breast, and other malignancies (Li et al., 2020). Previous study has demonstrated that TMB can be utilized as a biomarker to enhance the efficacy of immunotherapy (Yuan et al., 2022). Immunotherapy was useful for approximately 10 percent of cancer patients with high TMB (Chalmers et al., 2017). A genomic and proteomic examination of 232 cases of renal clear cell carcinoma revealed that NNMT contributes to the immune response of renal cell carcinoma *via* one-carbon metabolism and is a potential therapeutic target (Qu et al., 2022). Our research revealed that NNMT expression is related to MSI in seven cancer types and TMB in fourteen cancer types. This may suggest that the expression of NNMT influences the TMB and MSI of cancer, hence influencing the responsiveness of patients to immunosuppressive medication at immunological checkpoints.

We also paid attention to intracellular protein interactions, intracellular signaling pathways, and drug sensitivity, which is considered to be important. Enrichment analyses indicated that NNMT can potentially impact cell migration or motility, the PI3K/Akt pathway, protein metabolism, and multiple protein interactions. The expression of CDK2 and CDK6 was unregulated due to the overexpression of NNMT, while the expression of P53, P21, and P27 was downregulated, resulting in an imbalance between CDK and CDK inhibitor (Xie et al., 2014; Xie et al., 2016). NNMT is associated with the phosphorylation of kinases in the PI3K-Akt and MAPK-ERK pathways (Xie et al., 2014). In addition, silencing NNMT in gastric cancer cells significantly increased the proportion of cells in the G2 phase and inhibited cell activity (Chen et al., 2016).

Increasing research showed that aberrant DNA methylation may promote genomic instability *via* methylation modification silencing key anti-oncogenes, resulting in TME alterations, genomic instability, and biological process switching (Zhang et al., 2017). For instance, the degrees of promoter methylation of genes such as PTEN, FOXP3, and SIT1 have a major impact on their function (Bian et al., 2012; Ke et al., 2016). However, NNMT-induced alterations in methylation levels in a range of malignancies have not been described. We investigated the link between NNMT expression and promoter methylation because NNMT can change the methylation level of several cellular components. As expected, increased NNMT expression greatly decreased promoter methylation, which is strongly associated with poor prognosis.

**FIGURE 14:**
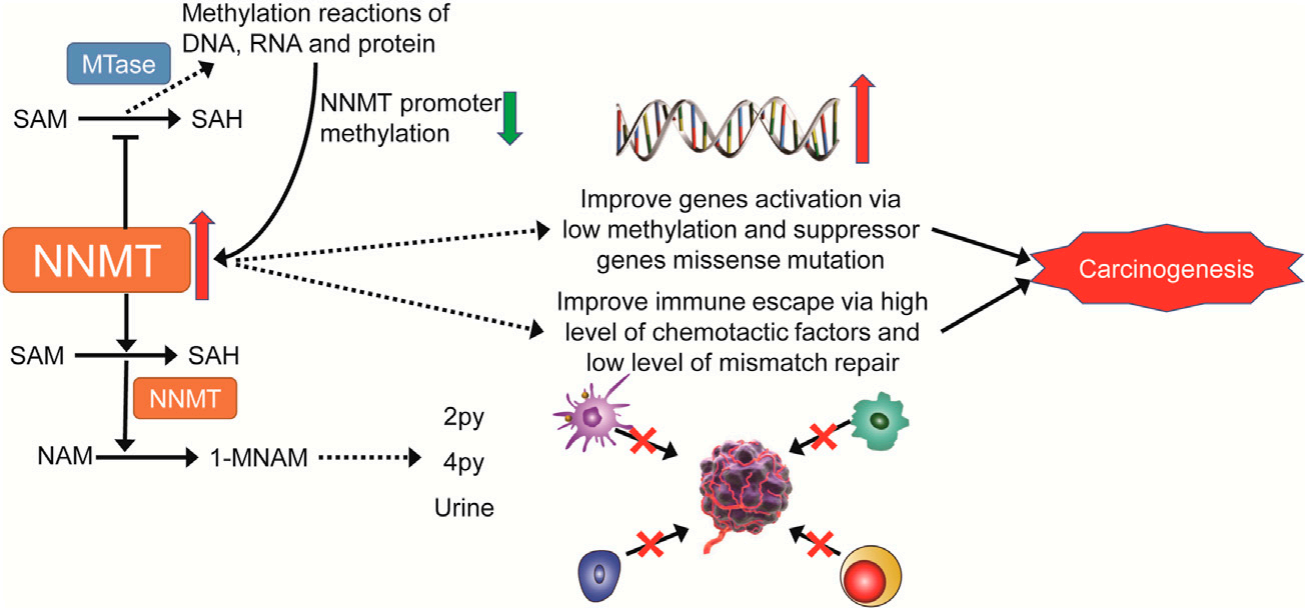
The multiple pathways through which NNMT may contribute to carcinogenesis are depicted in a mechanistic diagram.

In conclusion, our first pan-cancer investigation of NNMT demonstrated differential NNMT expression between tumors and normal tissues, as well as a link between NNMT expression and clinical prognosis and DNA methylation. Furthermore, there was a strong correlation between the expression of NNMT and immune cell infiltration of TMB, MSI, and other cancer types. Its effect on tumor immunity also varies by tumor type (Figure 14). Our findings indicate that NNMT can be employed as an independent prognostic factor for a variety of malignancies, and its expression level predicts a similar outcome for various tumors. It is necessary to conduct additional research into the unique function of NNMT in each form of cancer. These findings may help explain the role of NNMT in metabolism and tumor promotion, and provide a basis for future immune checkpoint inhibitors that are more targeted and individualized.

### Data collection

For RNA-seq data, 33 types cancer data with clinical information were downloaded from Xena including GDC TCGA Acute Myeloid Leukemia (LAML), GDC TCGA Adrenocortical Carcinoma (ACC), GDC TCGA Bile Duct Cancer (CHOL), GDC TCGA Bladder Cancer (BLCA), GDC TCGA Breast Cancer (BRCA), GDC TCGA Cervical Cancer (CESC), GDC TCGA Colon Cancer (COAD), GDC TCGA Endometrioid Cancer (UCEC), GDC TCGA Esophageal Cancer (ESCA), GDC TCGA Glioblastoma (GBM), GDC TCGA Head and Neck Cancer (HNSC), GDC TCGA Kidney Chromophobe (KICH), GDC TCGA Kidney Clear Cell Carcinoma (KIRC), GDC TCGA Kidney Papillary Cell Carcinoma (KIRP), GDC TCGA Large B-cell Lymphoma (DLBC), GDC TCGA Liver Cancer (LIHC), GDC TCGA Lower Grade Glioma (LGG), GDC TCGA Lung Adenocarcinoma (LUAD), GDC TCGA Lung Squamous Cell Carcinoma (LUSC), GDC TCGA Melanoma (SKCM), GDC TCGA Mesothelioma (MESO), GDC TCGA Ocular melanomas (UVM), GDC TCGA Ovarian Cancer (OV), GDC TCGA Pancreatic Cancer (PAAD), GDC TCGA Pheochromocytoma & Paraganglioma (PCPG), GDC TCGA Prostate Cancer (PRAD), GDC TCGA Rectal Cancer (READ), GDC TCGA Sarcoma (SARC), GDC TCGA Stomach Cancer (STAD), GDC TCGA Testicular Cancer (TGCT), GDC TCGA Thymoma (THYM), GDC TCGA Thyroid Cancer (THCA), GDC TCGA Uterine Carcinosarcoma (UCS) and GTEX. The FPKM value is converted to transcripts per kilobase million (TPM) values. The batch effect from non-biotech bias is corrected through the “normalizeBetweenArrays” function based on the “limma” R package. GTF files were obtained from Ensembl for annotation to identify mRNAs. Besides, we downloaded “CCLE_RNAseq_rsem_genes_tpm_2018.txt.gz”, “gencode.v19.genes.v7_model.patched_contigs.gtf.gz” and “Cell_lines_annotations_20181226.txt” files from CCLE database to analysis NNMT expression in cell lines.

For mutation data, 14 types cancer data were downloaded from TCGA including LGG, LUSC, LIHC, PAAD, SARC, SKCM, STAD, BLCA, HNSC, KIRP, UCEC, COAD, CESC and ESCA. MAF files were processed with “maftools” R package. Tumor samples were divided into two groups based on NNMT expression level. Top 20 mutation genes were showed with waterfall diagram.

For methylation data, 31 types cancer data including CESC, LGG, CHOL, KICH, KIRC, KIRP, LUAD, LUAD, PAAD, UCS, BLCA, ACC, BRCA, DLBC, ESCA, GBM, HNSC, LAML, LIHC, LUSC, MESO, PCPG, PRAD, SARC, SKCM, STAD, TGCT, THCA, THYM, UCEC, UVM, and COADREAD were accessed by “cgdsr” R package, which provides an API for cbioportal database. Methylation (HM27) and samples with mRNA data (RNA Seq V2) were extracted to study the relationship between promoter methylation and gene expression.

For protein data, five types cancer data including glioblastoma, clear cell renal cell carcinoma, uterine corpus endometrial carcinoma, lung adenocarcinoma, head and neck squamous cell carcinoma were downloaded from CPTAC to validate survival trend of NNMT.

### Genetic alteration analysis of nicotinamide n-methyltransferase

The cBioPortal (version: 3.6.20) (https://www.cbioportal.org/) and 33 TCGA tumors somatic mutation data sets were used to assess NNMT gene mutations. In the “Summary” section, we computed the number of NNMT gene mutation types and copy number alterations. A mutation site plot for NNMT was generated in the “Mutation” module. Somatic mutation data categorized by NNMT expression was presented as waterfall charts in 14 malignancies based on TCGA data. The Chi-square test was used to compare the frequency of gene mutations in each sample group (*p* < 0.05 was considered significant).

### Association between nicotinamide n-methyltransferase expression and mismatch repair genes, microsatellite instability and tumor mutational burden

Mismatch Repair (MMR) was an intracellular mechanism for DNA repair. Down-regulation or functional problems in the MMR genes could result in irreparable DNA replication errors, leading to an increase in the incidence of somatic mutations. TCGA expression profile data were used to evaluate the expression levels of MMR genes, such as MutL homologous 1 (MLH1), MutS homologous 2 (MSH2), MutS homologous 3 (MSH3), MutS homologous 6 (MSH6), postmeiotic segregation increased 1 (PMS1), postmeiotic segregation increased 2 (PMS2), and epithelial cell adhesion molecule (EPCAM) displayed as heat maps created with the R packages “reshape2” and “RColorBrewer."

MSI represented the amount of repeated DNA bases in a microsatellite (a short, repetitive sequence of DNA) and was a crucial predictor of the incidence and growth of malignancies (Li et al., 2020). TMB was a numeric measure that indicated a potential immunotherapy response based on the number of mutations per million bases (muts/Mb) carried by cancer cells in a tumor (Lawlor et al., 2021). On the basis of somatic mutation data from TCGA, MSI scores were calculated for all samples, and the association between NNMT expression and TMB and MSI was evaluated using Spearman’s rank correlation. To use a Perl script, TMB scores were computed and afterwards corrected by dividing by the total length of exons. Using the R package “fmsb”, radar charts were produced to display the results.

### nicotinamide n-methyltransferase sites for epigenetic modification and somatic mutation

The UCSC genome browser (GRCh38/hg38) (https://genome.ucsc.edu/index.html) was used to visualize gene epigenetic modification and somatic mutation sites of NNMT.

## Data Availability

The datasets presented in this study can be found in online repositories. The names of the repository/repositories and accession number(s) can be found in the article/Supplementary Material.

## Glossary

ACC: Adrenocortical carcinoma
BLCA: Bladder Urothelial Carcinoma
BP: Biological progress
BRCA: Breast invasive carcinoma
CC: Cellular component
CCLE: Cancer cell line encyclopedia
CESC: Cervical squamous cell carcinoma and endocervical adenocarcinoma
CHOL: Cholangiocarcinoma
COAD: Colon adenocarcinoma
COADREAD: Colon adenocarcinoma/Rectum adenocarcinoma Esophageal carcinoma
CPTAC: Clinical Proteomic Tumor Analysis Consortium
DFS: Disease free survival
DLBC: Diffuse Large B-cell Lymphoma
EMT: Epithelial-Mesenchymal Transition
ESCA: Esophageal carcinoma
FDA: Food and Drug Administration
GEPIA: Gene Expression Profiling Interactive Analysis
GTEx: Genotype-Tissue Expression
HNSC: Head and Neck squamous cell carcinoma
IHC: Immunohistochemistry
KICH: Kidney chromophobe
KIRC: Kidney renal clear cell carcinoma
KIRP: Kidney renal papillary cell carcinoma
LAML: Acute Myeloid Leukemia
LGG: Lower Grade Glioma
LIHC: Liver Hepatocellular carcinoma
LUAD: Lung Adenocarcinoma
LUSC: Lung squamous cell carcinoma
MF: Molecular function
MMR: Mismatch repair
MSI: Microsatellite instability
OV: Ovarian serous cystadenocarcinoma
PAAD: Pancreatic Aadenocarcinoma
PPI: Protein-protein interaction
PRAD: Prostate Adenocarcinoma
SARC: Sarcoma
SKCM: Skin Cutaneous Melanoma
STAD: Stomach adenocarcinoma
STES: Stomach and Esophageal carcinoma
TCGA: The Cancer Genome Atlas
TGCT: Testicular Germ Cell Tumors
THCA: Thyroid carcinoma
THYM: Thymoma
TIMER: Tumor Immune Estimation Resource
TMB: Tumor Mutational Burden
TME: Tumor microenvironment
UCEC: Uterine Corpus Endometrial Carcinoma
UCS: Uterine Carcinosarcoma
UVM: Uveal Melanoma
