# Supplementary material for: Systematic pan-cancer analysis of the nicotinamide n-methyltransferase in human cancer
Source: Front Genet. 2022 Oct 26;13:1000515. doi: 10.3389/fgene.2022.1000515 (PMC9644023; doi:10.3389/fgene.2022.1000515)
Supplement: Supplementary file 2 [file Table1.DOCX]

## 2.1 Data Collection

For RNA-seq data, 33 types cancer data with clinical information were downloaded from Xena including GDC TCGA Acute Myeloid Leukemia (LAML), GDC TCGA Adrenocortical Cancer (ACC), GDC TCGA Bile Duct Cancer (CHOL), GDC TCGA Bladder Cancer (BLCA), GDC TCGA Breast Cancer (BRCA), GDC TCGA Cervical Cancer (CESC), GDC TCGA Colon Cancer (COAD), GDC TCGA Endometrioid Cancer (UCEC), GDC TCGA Esophageal Cancer (ESCA), GDC TCGA Glioblastoma (GBM), GDC TCGA Head and Neck Cancer (HNSC), GDC TCGA Kidney Chromophobe (KICH), GDC TCGA Kidney Clear Cell Carcinoma (KIRC), GDC TCGA Kidney Papillary Cell Carcinoma (KIRP), GDC TCGA Large B-cell Lymphoma (DLBC), GDC TCGA Liver Cancer (LIHC), GDC TCGA Lower Grade Glioma (LGG), GDC TCGA Lung Adenocarcinoma (LUAD), GDC TCGA Lung Squamous Cell Carcinoma (LUSC), GDC TCGA Melanoma (SKCM), GDC TCGA Mesothelioma (MESO), GDC TCGA Ocular melanomas (UVM), GDC TCGA Ovarian Cancer (OV), GDC TCGA Pancreatic Cancer (PAAD), GDC TCGA Pheochromocytoma & Paraganglioma (PCPG), GDC TCGA Prostate Cancer (PRAD), GDC TCGA Rectal Cancer (READ), GDC TCGA Sarcoma (SARC), GDC TCGA Stomach Cancer (STAD), GDC TCGA Testicular Cancer (TGCT), GDC TCGA Thymoma (THYM), GDC TCGA Thyroid Cancer (THCA), GDC TCGA Uterine Carcinosarcoma (UCS) and GTEX. The FPKM value is converted to transcripts per kilobase million (TPM) values. The batch effect from non-biotech bias is corrected through the “normalizeBetweenArrays” function based on the “limma” R package. GTF files were obtained from Ensembl for annotation to identify mRNAs. Besides, we downloaded “CCLE_RNAseq_rsem_genes_tpm_2018.txt.gz”, “gencode.v19.genes.v7_model.patched_contigs.gtf.gz” and “Cell_lines_annotations_20181226.txt” files from CCLE database to analysis NNMT expression in cell lines.

For mutation data, 14 types cancer data were downloaded from TCGA including LGG, LUSC, LIHC, PAAD, SARC, SKCM, STAD, BLCA, HNSC, KIRP, UCEC, COAD, CESC and ESCA. MAF files were processed with “maftools” R package. Tumor samples were divided into two groups based on NNMT expression level. Top 20 mutation genes were showed with waterfall diagram.

For methylation data, 31 types cancer data including CESC, LGG, CHOL, KICH, KIRC, KIRP, LUAD, LUAD, PAAD, UCS, BLCA, ACC, BRCA, DLBC, ESCA, GBM, HNSC, LAML, LIHC, LUSC, MESO, PCPG, PRAD, SARC, SKCM, STAD, TGCT, THCA, THYM, UCEC, UVM and COADREAD were accessed by “cgdsr” R package, which provides an API for cbioportal database. Methylation (HM27) and samples with mRNA data (RNA Seq V2) were extracted to study the relationship between promoter methylation and gene expression.

For protein data, 5 types cancer data including glioblastoma, clear cell renal cell carcinoma, uterine corpus endometrial carcinoma, lung adenocarcinoma, head and neck squamous cell carcinoma were downloaded from CPTAC to validate survival trend of NNMT.

## 2.2 Genetic Alteration Analysis of NNMT

The cBioPortal (version: 3.6.20) (https://www.cbioportal.org/) and 33 TCGA tumors somatic mutation data sets were used to assess NNMT gene mutations. In the "Summary" section, we computed the number of NNMT gene mutation types and copy number alterations. A mutation site plot for NNMT was generated in the "Mutation" module. Somatic mutation data categorized by NNMT expression was presented as waterfall charts in 14 malignancies based on TCGA data. The Chi-square test was used to compare the frequency of gene mutations in each sample group (P < 0.05 was considered significant).

## 2.3 Association between NNMT Expression and Mismatch Repair Genes, MSI and TMB

Mismatch Repair (MMR) was an intracellular mechanism for DNA repair. Down-regulation or functional problems in the MMR genes could result in irreparable DNA replication errors, leading to an increase in the incidence of somatic mutations. TCGA expression profile data were used to evaluate the expression levels of MMR genes, such as MutL homologous 1 (MLH1), MutS homologous 2 (MSH2), MutS homologous 3 (MSH3), MutS homologous 6 (MSH6), postmeiotic segregation increased 1 (PMS1), postmeiotic segregation increased 2 (PMS2), and epithelial cell adhesion molecule (EPCAM) displayed as heat maps created with the R packages "reshape2" and "RColorBrewer."

MSI represented the amount of repeated DNA bases in a microsatellite (a short, repetitive sequence of DNA) and was a crucial predictor of the incidence and growth of malignancies (Li et al., 2020). TMB was a numeric measure that indicated a potential immunotherapy response based on the number of mutations per million bases (muts/Mb) carried by cancer cells in a tumor(Lawlor et al., 2021). On the basis of somatic mutation data from TCGA, MSI scores were calculated for all samples, and the association between NNMT expression and TMB and MSI was evaluated using Spearman's rank correlation. To use a Perl script, TMB scores were computed and afterwards corrected by dividing by the total length of exons. Using the R package “fmsb”, radar charts were produced to display the results.

## 2.4 NNMT Sites for epigenetic modification and somatic mutation

The UCSC genome browser (GRCh38/hg38) (https://genome.ucsc.edu/index.html) was used to visualize gene epigenetic modification and somatic mutation sites of NNMT.

# References

LAWLOR, R. T., MATTIOLO, P., MAFFICINI, A., HONG, S. M., PIREDDA, M. L., TAORMINA, S. V., MALLEO, G., MARCHEGIANI, G., PEA, A., SALVIA, R., KRYKLYVA, V., SHIN, J. I., BROSENS, L. A., MILELLA, M., SCARPA, A. & LUCHINI, C. 2021. Tumor Mutational Burden as a Potential Biomarker for Immunotherapy in Pancreatic Cancer: Systematic Review and Still-Open Questions. *Cancers (Basel),* 13.

LI, K., LUO, H., HUANG, L., LUO, H. & ZHU, X. 2020. Microsatellite instability: a review of what the oncologist should know. *Cancer Cell Int,* 20**,** 16.
