# Supplementary figures and images for: Systematic pan-cancer analysis of the nicotinamide n-methyltransferase in human cancer
Source: Front Genet. 2022 Oct 26;13:1000515. doi: 10.3389/fgene.2022.1000515 (PMC9644023; doi:10.3389/fgene.2022.1000515)

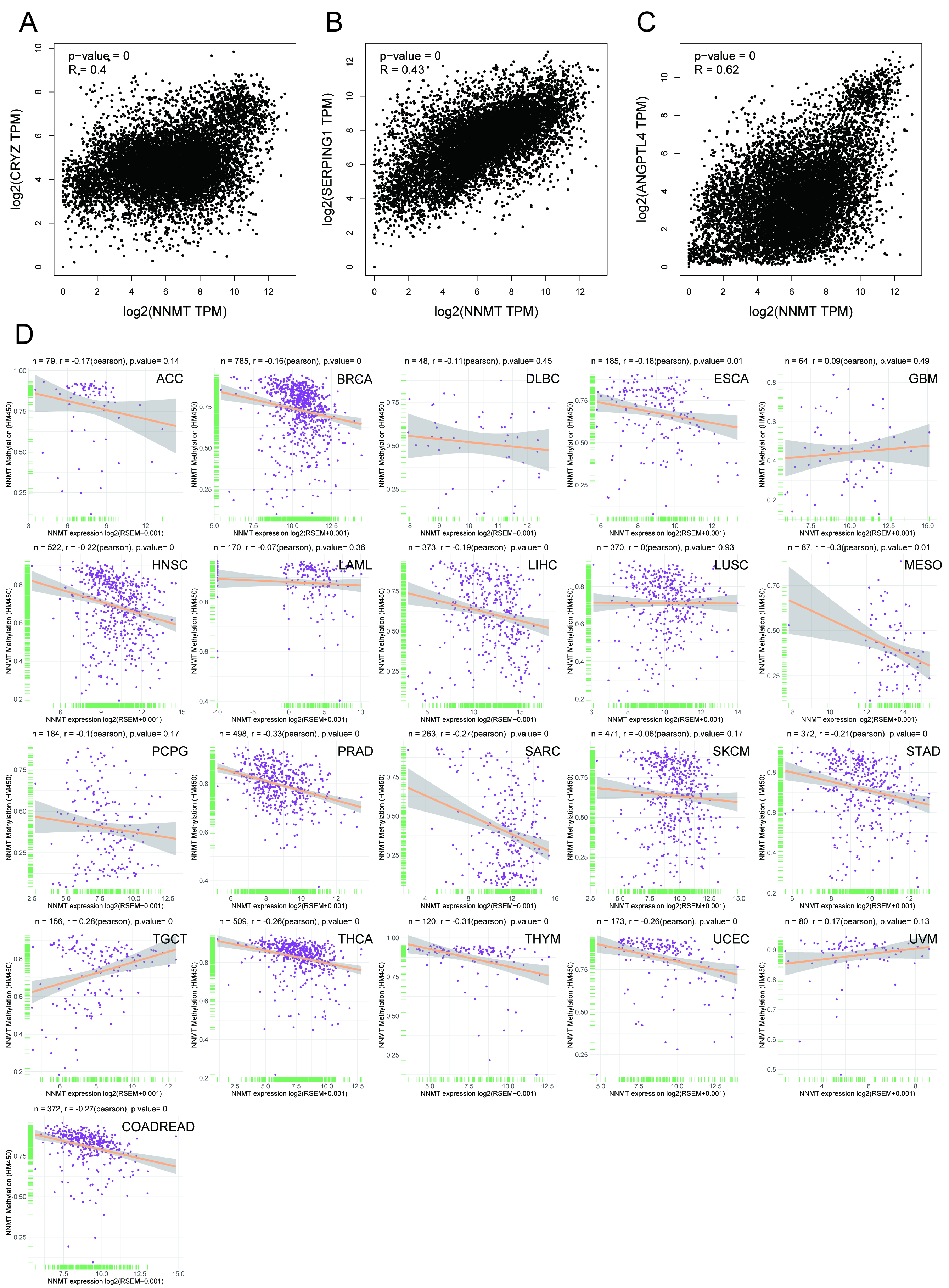

Supplement: Supplementary file 3 [file Image3.TIF]

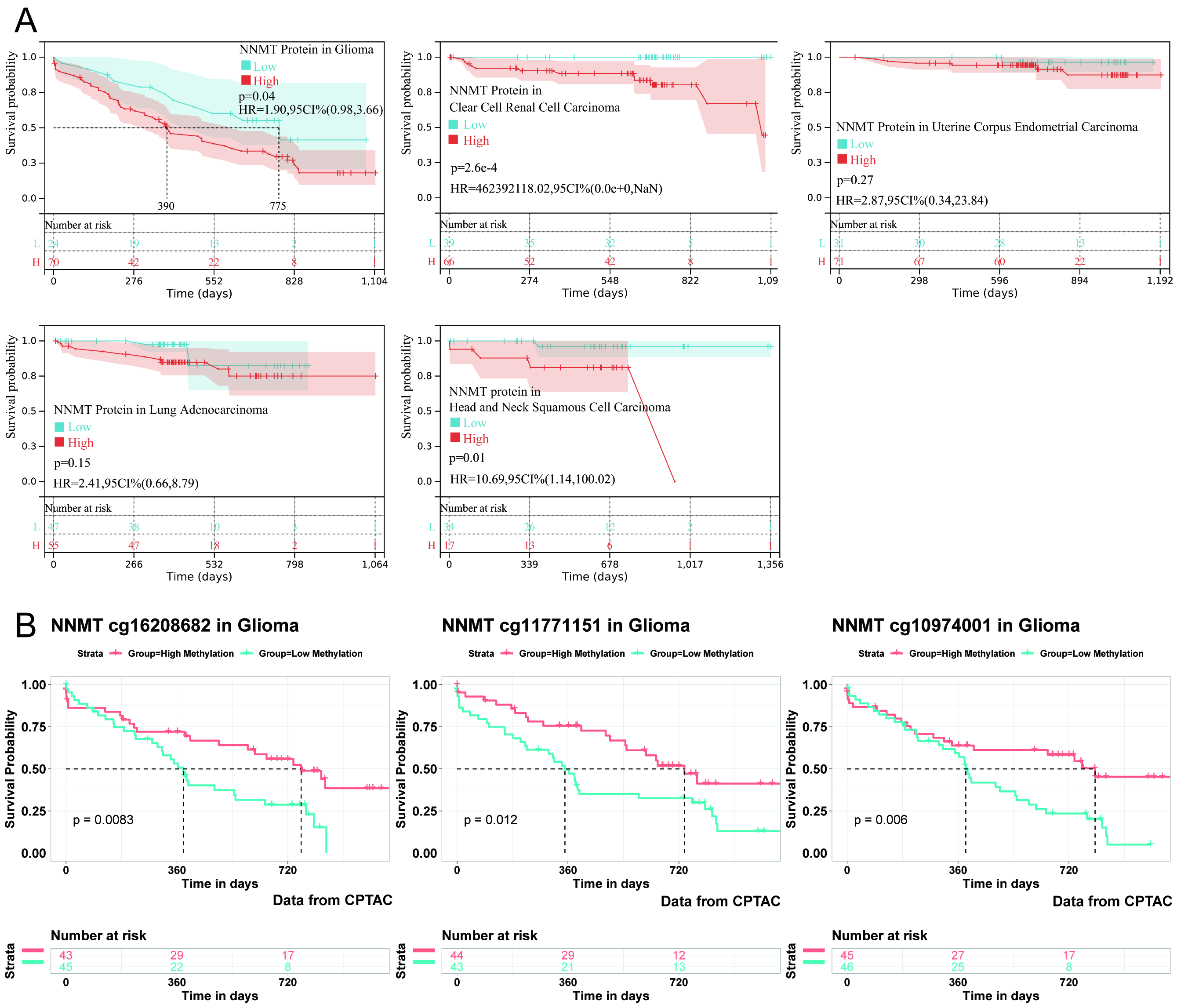

Supplement: Supplementary file 4 [file Image4.TIF]

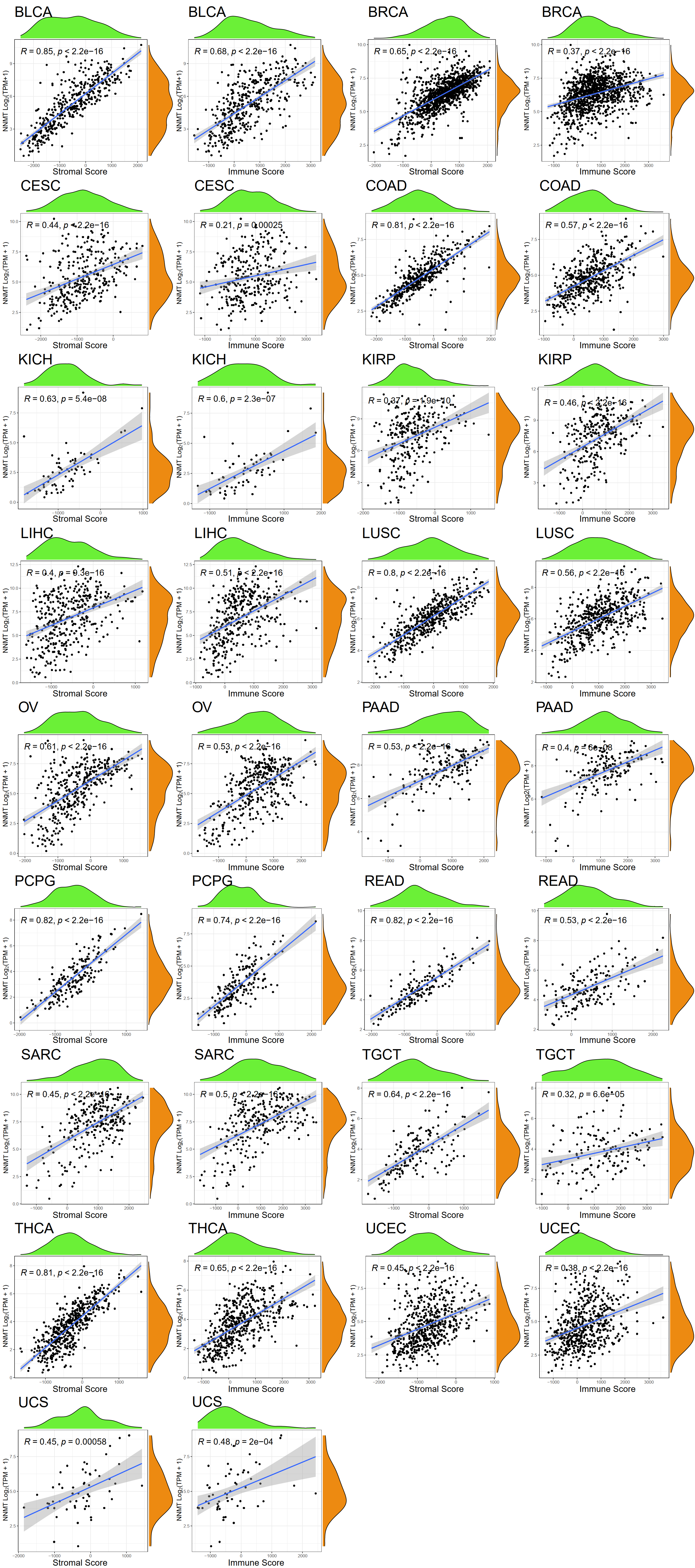

Supplement: Supplementary file 6 [file Image1.TIF]
